# Enhanced germline stem cell longevity in *Drosophila* diapause

**DOI:** 10.1038/s41467-022-28347-z

**Published:** 2022-02-07

**Authors:** Sreesankar Easwaran, Matthew Van Ligten, Mackenzie Kui, Denise J. Montell

**Affiliations:** Molecular, Cellular, and Developmental Biology Department, University of California, Santa Barbara, CA 93106 USA

**Keywords:** Ageing, Senescence, Reproductive biology, Quiescence

## Abstract

In many species including humans, aging reduces female fertility. Intriguingly, some animals preserve fertility longer under specific environmental conditions. For example, at low temperature and short day-length, *Drosophila melanogaster* enters a state called adult reproductive diapause. As in other stressful conditions, ovarian development arrests at the yolk uptake checkpoint; however, mechanisms underlying fertility preservation and post-diapause recovery are largely unknown. Here, we report that diapause causes more complete arrest than other stresses yet preserves greater recovery potential. During dormancy, germline stem cells (GSCs) incur DNA damage, activate p53 and Chk2, and divide less. Despite reduced niche signaling, germline precursor cells do not differentiate. GSCs adopt an atypical, suspended state connected to their daughters. Post-diapause recovery of niche signaling and resumption of division contribute to restoring GSCs. Mimicking one feature of quiescence, reduced juvenile hormone production, enhanced GSC longevity in non-diapausing flies. Thus, diapause mechanisms provide approaches to GSC longevity enhancement.

## Abstract (web summary)

Drosophila enter adult reproductive diapause in low temperatures and short day, halting ovarian development yet preserving fertility. Here the authors show that ovarian arrest in diapause is distinct from other stress responses and that despite DNA damage and decreased division, germline stem cells recover.

## Introduction

Important clues to healthy human aging have come from studying the natural ability of simple, relatively short-lived animals to enter into long-lived, stress-resistant states^1,2^. Many organisms enter a state of dormancy in response to environmental cues, which allows survival and a return to reproduction when conditions improve. These states include diapause, dauer, and hibernation, among others^3–6^. Different species enter dormancy optimally at distinct life stages and in response to various stresses such as high or low temperature, desiccation, crowding, or starvation. The fruit fly *Drosophila melanogaster* enters a quiescent state when animals experience cool temperatures (10–12 °C) and short day length (12–16 h of darkness) simulating fall^7,8^. The optimal stage in *Drosophila* is the newly eclosed adult.

Like other dormancy states, adult reproductive diapause in *Drosophila* is characterized by reduced motor activity, altered metabolism and physiology, increased stress tolerance, and cessation of reproduction^9–14^. While *Drosophila* diapause depends on the temperature to a greater degree than day length and is sometimes referred to as temperature-dependent quiescence or dormancy, there is extensive literature that refers to it as diapause^8,9,15–22^, as we do here. Furthermore, hormonal regulation of the program has been studied^16,17^, and like *Caenorhabditis elegans* dauer, *Drosophila* diapause is regulated by insulin receptor signaling^15,17–22^.

While global changes in endocrine signaling, metabolism, and gene expression during *Drosophila* adult reproductive diapause have been documented^9,11,15,23–25^, mechanisms that preserve fertility and drive post-diapause recovery are largely unknown. Elucidating the mechanisms that support dormancy may reveal strategies for maintaining youthful states, including fertility, during aging^11^. Here, we report the effects of adult reproductive diapause on *Drosophila* germline stem cells (GSCs) and provide insights into the cellular and molecular mechanisms that preserve female reproductive potential.

## Results

### Diapause enhances ovarian longevity

**Fig. 1:**
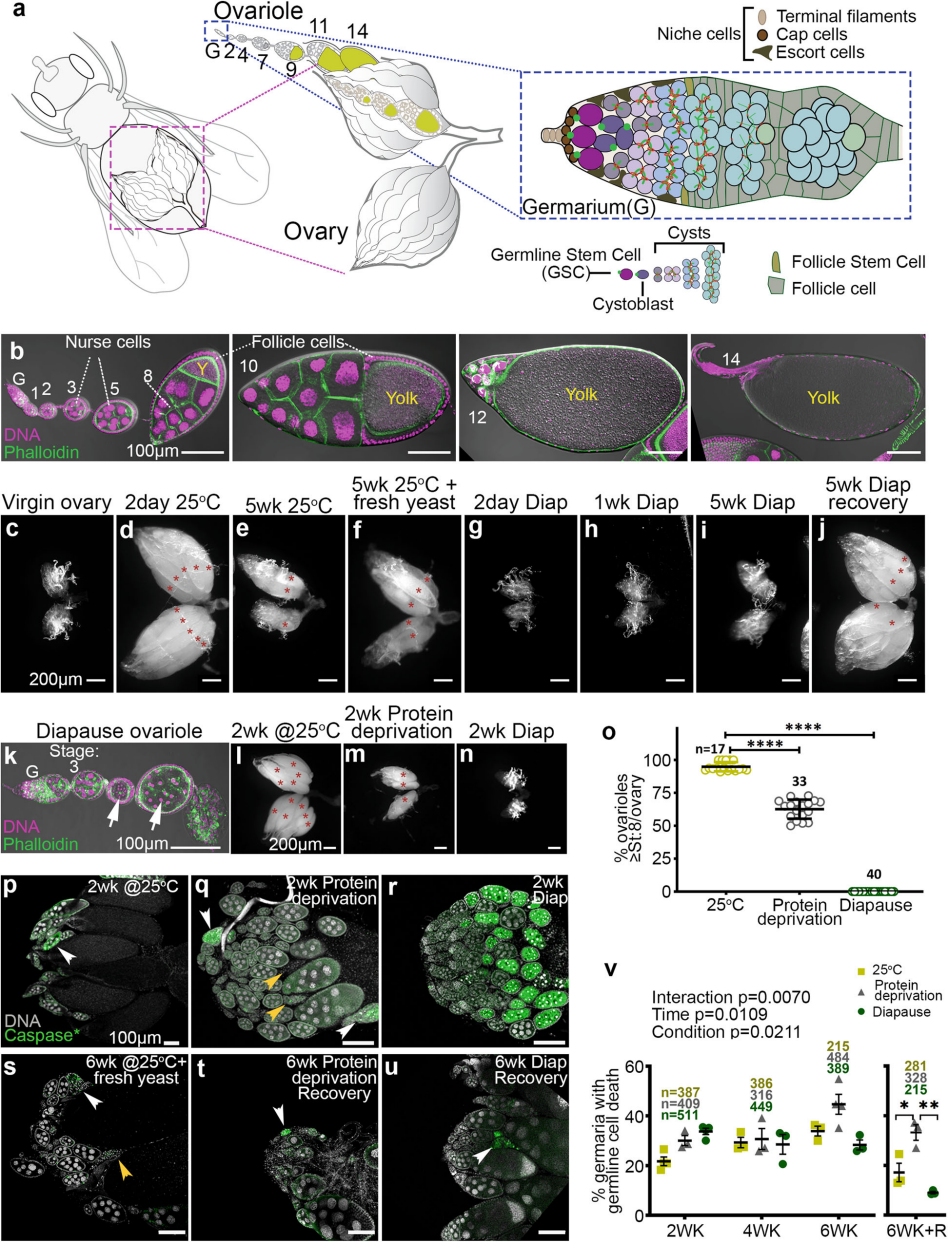
Distinct ovarian arrest and recovery responses to diapause and other stresses. **a** Schematic drawings of ovarian anatomy at increasing magnifications (left to right). Yellow indicates yolk. **b** Confocal images showing stages of normal egg chamber development. Scale bars 100 µm. **c**–**j** Darkfield micrographs of whole ovaries for the indicated conditions showing normal development, aging, diapause, and recovery. Asterisks denote mature eggs. Scale bars 200 µm. **k** Degenerating early stage egg chambers (white arrows) during diapause. Scale bar 100 µm. **l**–**n** Darkfield micrographs of whole ovaries from flies grown under the indicated conditions. Asterisks label mature eggs. Scale bars 200 µm. **o** Percentage of ovarioles with egg chambers that developed beyond the yolk deposition checkpoint. *n* is the number of ovaries analyzed and data are mean ± s.d.; ****p < 0.0001 (1-way ANOVA and Tukey’s multiple comparison test). **p**–**u** Micrographs of anti-active caspase staining (green) of whole ovaries from the indicated conditions. Examples of caspase-positive, degenerating egg chambers (white arrowheads) or caspase-negative, fully degenerated egg chambers (yellow arrowheads). Scale bars 100 µm. **v** Comparison of the germline cell death in the germaria from flies during normal development and aging, subjected to protein deprivation, or in diapause and recovery (R). Data are means from at least three independent experiments ± s.e.m. Numbers (*n*) of germaria counted. Statistical analysis using 2-way ANOVA was carried out before the recovery time period. After recovery, Tukey’s multiple comparisons test and 1-way ANOVA test was conducted (*p = 0.0162; **p = 0.0022).

To gain insight into the mechanisms that preserve fertility, we examined all stages of ovarian development during diapause and recovery. Fly ovaries are composed of ovarioles, which are strands of developing eggs (Fig. 1a). At the anterior tip, in a structure called the germarium, there is a cellular niche composed of terminal filament, cap, and escort cells, which support 2–3 GSCs. When GSCs divide, they self-renew and produce a daughter cystoblast, which undergoes four additional rounds of division with incomplete cytokinesis to form a 16-cell cyst (Fig. 1a). Continuous production of new cystoblasts and cysts pushes older cysts posteriorly. Somatic cells envelop each cyst to form egg chambers, which grow and develop through 14 stages to produce mature eggs (Fig. 1b). From stage 8, vitellogenic egg chambers take up yolk.

Ovaries of newly eclosed females are small and contain only previtellogenic egg chambers (Fig. 1c). When maintained under optimal growth conditions at 18–25 °C, with 12 h of light and 12 h of dark (12L:12D) for 2 days, egg chambers grow and develop and fill the ovary with all stages, including mature eggs (Fig. 1d). When flies were aged for 5 weeks at 25 °C, the ovaries senesced and shrank (Fig. 1e). If the fly food was then supplemented with fresh yeast, a few egg chambers matured, enlarging the ovary slightly (Fig. 1f). By contrast, in flies maintained in diapause-inducing conditions [10 °C with 8L:16D^26,27^], little to no ovarian growth occurred over 5 weeks (Fig. 1g–i). Diapause arrest of oogenesis was 100% penetrant in these conditions. Strikingly, when these flies were moved to 18 °C for one day followed by one day at 25 °C and in the presence of fresh yeast (recovery conditions), the ovaries filled with egg chambers of all stages (Fig. 1j), similar to young, non-diapausing flies (Fig. 1d). Ninety-four percent of females recovered fertility after 5 weeks in diapause. Both fertility (% of females that produce any offspring) and fecundity (the number of viable offspring per female) declined with longer times in diapause (Table 1). We used 5–6 weeks of diapause for the remainder of our studies.

**Table 1:** Four-day fecundity and fertility in non-diapause and post-diapause female flies.

Condition	*n*	Mean fecundity	% Fertility
Non-diapause	11	66.9	100
5WK diapause + Recovery	16	22.5	93.8
9WK diapause + Recovery	9	3.4	33.3

Entry into *Drosophila* diapause is known to be more sensitive to temperature than to day length^28^. To assess the relative importance of these two variables on ovarian arrest and recovery, we compared the responses of flies maintained at 10 °C in a long day (16L:8D) to short day (8L:16D) conditions (Supplementary Fig. 1). Compared to standard short day conditions, the long day allowed more ovarian development and thus larger ovaries compared to short day flies at week 2 (Supplementary Fig. 1a–c). The effects of the two conditions were indistinguishable at weeks 4 and 6, suggesting that the long day had delayed the entry into diapause. We conclude that day length has a small but detectable effect.

### Diapause arrest is distinct from other stress responses

During diapause, egg chamber development arrests at stage 7, at the yolk uptake checkpoint^8,14^, similar to other environmental stresses such as protein deprivation^29–31^ or exposure to parasitoid wasps^32^. The effects of diapause on earlier stages of oogenesis have not been reported. In ovaries from diapausing flies, we noted degenerating early-stage egg chambers, from ~stage 4 onward, indicated by condensed nurse cell nuclei (Fig. 1k, arrows), in addition to stage 7 arrest.

Diapausing flies consume less food^9^, suggesting that they could be nutrient-limited, though circulating carbohydrates and lipids are actually elevated^9^. Since protein deprivation also disrupts oogenesis^33^, we compared the effects of diapause to those of protein deprivation. Compared to ovaries from 2-week-old flies cultured in optimal growth conditions (Fig. 1l), ovaries from protein-deprived flies were smaller and contained fewer mature eggs (Fig. 1m). However, they were not as under-developed as ovaries from diapausing flies (Fig. 1n). Totally, 95% of ovarioles from well-fed females kept in non-diapausing conditions contained stage 8 and older egg chambers, compared to 63% for protein-deprived flies, and 0% for diapausing flies (Fig. 1o). Egg chamber degeneration and cell death, detected by staining for activated executioner caspase, were rare in ovaries from well-fed females in optimal growth conditions (Fig. 1p), which contained many stage 14 eggs. In contrast, protein deprivation causes germline cell death and egg chamber degeneration at two stages of development: germarium region 2 and stage 8^30^ (Fig. 1q). More extensive cell death and egg chamber degeneration occurred during diapause compared to protein deprivation (Fig. 1r). However, upon transfer to optimal growth conditions, post-diapause ovarian development recovered better, and there was less egg chamber degeneration and cell death than in flies that had been maintained at 25 °C either on protein-rich or protein-poor food (Fig. 1s–v). Thus, even though diapause causes a more complete arrest of ovarian development than protein deprivation, the capacity to recover is preserved better.

We further compared the ovarian response to diapause to two additional stress conditions: predator exposure and high temperature. When flies are exposed to the parasitoid wasp *Leptopilina heterotoma* (strain Lh14), they perceive a threat and exhibit reduced fecundity, presumably to prevent futile progeny production^32^. Wasp-exposed flies hold mature eggs within the ovary and exhibit egg chamber degeneration at the stage 8 yolk deposition checkpoint but not at earlier stages^32^. In contrast to diapausing flies, wasp-exposed flies exhibit an increase in ovary size, compared to unexposed, non-diapausing flies, due to the held eggs (Supplementary Fig. 1d–j). Together these data suggest that diapause, protein deprivation, and predator exposure produce distinct effects on early ovarian development despite activating the common stage 7/8 yolk deposition checkpoint.

To ascertain how the cold stress of diapause compares to another temperature stress, we examined the effects of heat stress on ovarian development. Ovaries from flies maintained at 30 °C were indistinguishable from 25 °C (Supplementary Fig. 1k–l'), although lifespan was shortened: 100% died within 4 weeks (*n* = 50) compared to 7% at 25 °C and <1% at 10 °C. At 33 °C, 100% of flies died within 1 week (*n* = 113). Ovaries dissected from 33 °C, 2-day-old survivors were small (Supplementary Fig. 1m), but nearly half contained at least one mature egg (Supplementary Fig. 1m'), demonstrating that ovarian arrest was less complete than diapause (Supplementary Fig. 1n, n’). At even higher temperatures, flies died within hours, although it is possible that varying the temperature to mimic daytime warming and nighttime cooling would be tolerated better. We conclude that high and low temperatures have different effects, with 10 °C producing a more complete arrest of ovarian development and better survival.

### Multistage diapause arrest

**Fig. 2:**
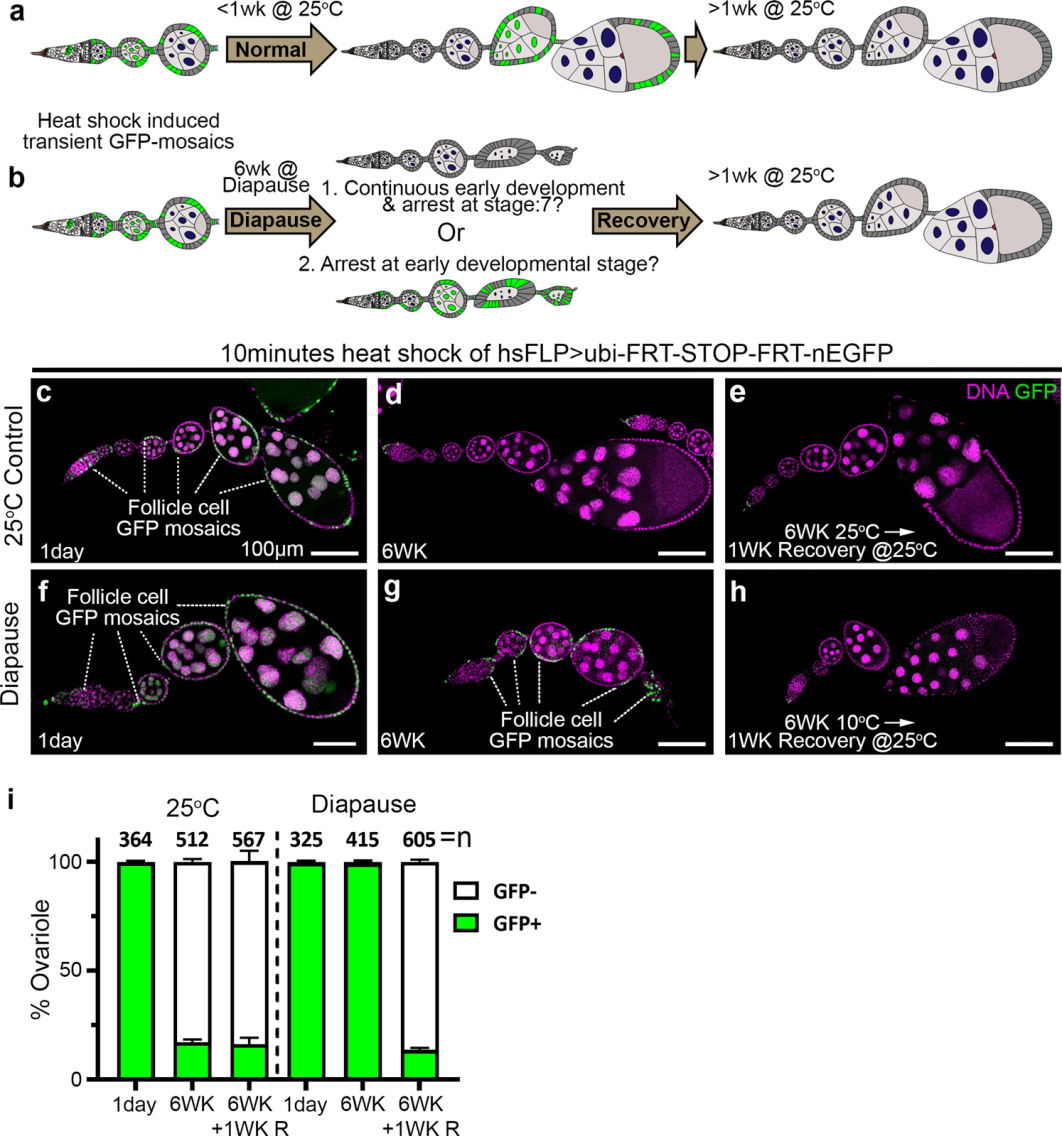
Diapause arrest at multiple stages. **a**, **b** Schematic representation of the experimental design. Transient, GFP + clones are generated by heat shock (see methods for details). **a** As egg chambers develop, those containing GFP + cells mature into eggs that are laid, and are replaced by unlabeled egg chambers. **b** In diapause, if egg chambers are continually produced and degenerating (option 1, top), GFP labeling would be lost, but if egg chamber development stops (option 2, bottom), degenerating GFP-labeled egg chambers would persist. In either case, upon transfer to recovery conditions, development should resume and GFP labeling should disappear. **c**–**e** Controls showing the presence of transient GFP clones 1 day after heat shock (**c**) and loss thereafter (**d**, **e**). **f**–**h** In diapause, GFP-labeled egg chambers persist for six weeks and are lost after a week of recovery. Scale bars 100 µm. **i** Quantification of the percentage of ovarioles containing any egg chamber younger than stage 14 with GFP + cells, comparing normal aging to diapause and recovery. The data shown are from three independent experiments. Error bars = mean ± s.e.m. *n* is the total number of ovarioles analyzed. See also Supplementary Fig. 2.

We reasoned that the stage 4–7 egg chamber degeneration observed during diapause (Fig. 1k) could either arise by: (1) continuous production of new cysts accompanied by continuous egg chamber degeneration and clearance; or (2) early arrest and slow degeneration. To distinguish between these two possibilities, we carried out lineage tracing, shown schematically in Fig. 2a, b. We induced transient mitotic recombination, resulting in mosaic green fluorescent protein (GFP) expression (Fig. 2a–c), and followed the fates of labeled egg chambers over time. Under normal growth conditions, egg chambers containing GFP-positive cells mature, eventually producing eggs that are laid (Fig. 2a). Therefore, GFP labeling should be lost after ~1 week at 25 °C (or 2 weeks at 18 °C), which is the time required for egg chamber maturation. During diapause, if egg chambers were continuously produced and degenerating, GFP would also be lost over time (Fig. 2b, option 1). In contrast, if new egg chamber production stops, then we would expect GFP-labeled egg chambers to persist (Fig. 2b, option 2). As expected, under normal growth conditions, GFP-labeled egg chambers were present 1 day after clone induction by heat shock (Fig. 2c and Supplementary Fig. 2a–c), were lost after 1 week at 25 °C (Supplementary Fig. 2d–f), and remained absent thereafter (Fig. 2d, e). In contrast to the control, GFP-positive egg chambers persisted through 6 weeks of diapause (Fig. 2f, g), consistent with early developmental arrest (option 2). During recovery from diapause, GFP-labeled egg chambers were replaced by newly formed, GFP-negative egg chambers within 1 week (Fig. 2h, i). We conclude that diapause arrests early stages of ovarian development, in addition to the well-established yolk deposition checkpoint.

### GSCs in diapause arrest and recovery

**Fig. 3:**
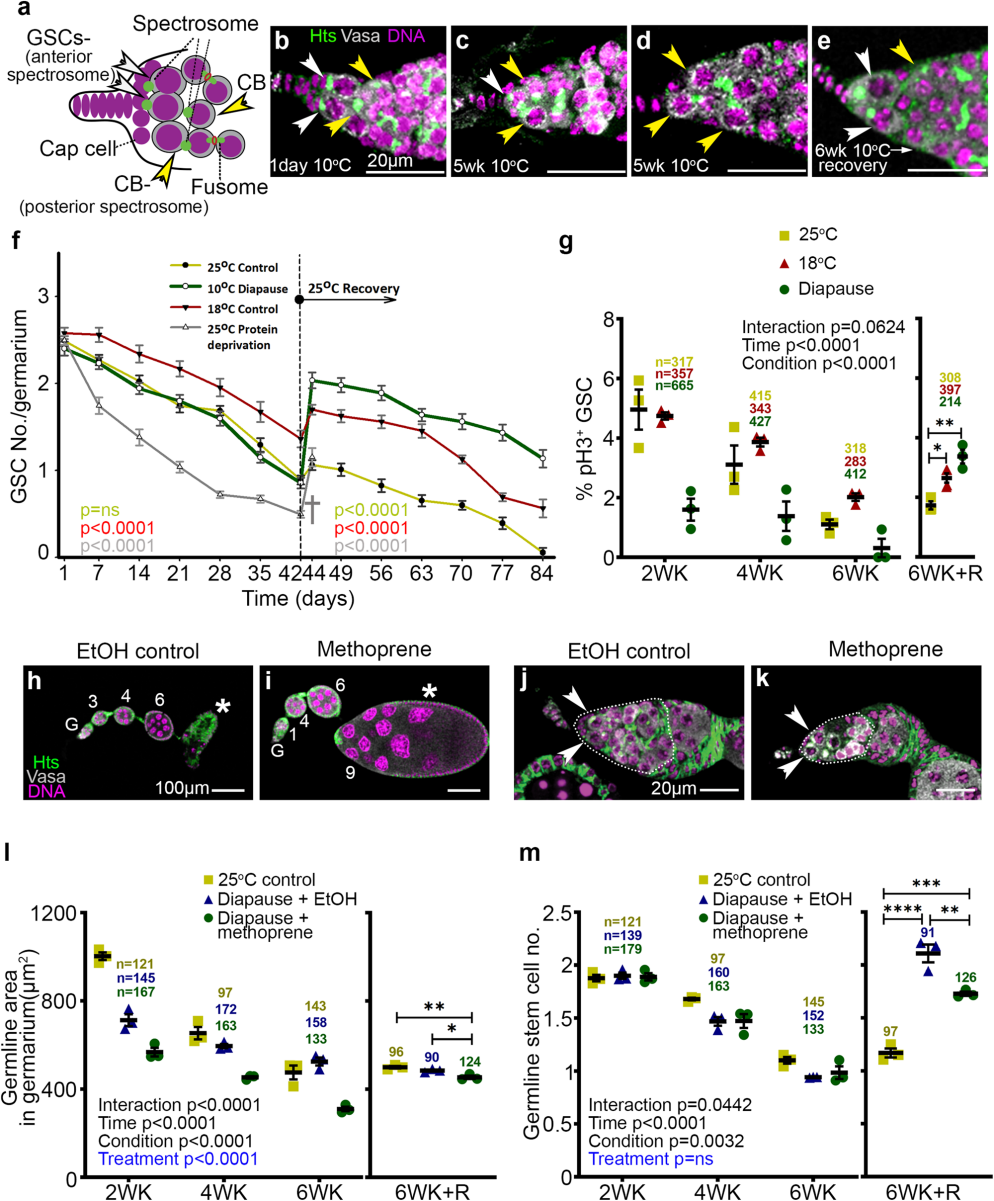
Germline stem cells in diapause arrest and recovery. **a** Schematic of a germarium tip showing GSCs (white arrowheads) in contact with niche cap cells and containing an anterior (left) spectrosome and a cystoblast (yellow arrowhead) with a posterior spectrosome and/or separated from the niche. **b**–**e** Confocal micrographs of representative germaria from diapause and recovery are stained with Vasa (white), Hts (green), and Hoechst (magenta). Nondiapause germaria are shown in Supplementary Fig. 3a–d. Scale bars 20 µm. **f** Quantification of GSC numbers over six weeks in the indicated conditions (control, diapause, protein deprivation) followed by 6 weeks at 25 °C. GSC numbers declined during diapause (green line) and rebounded during recovery. Protein deprivation led to premature death (†) so GSC data were not available after 44 days. Data = mean ± s.e.m. and refer to Supplementary Fig. 3e which has the *n*, number of germaria analyzed. *p* value for the condition was calculated using 2-way ANOVA (carried out separately for before and after the recovery time period) individually for each condition. **g** Percentage of GSCs in mitosis, assessed using anti-pH3 staining, over six weeks (WK) in the indicated conditions, followed by recovery (R). (*n*) number of GSCs analyzed. *p = 0.0179; **p = 0.0011. **h**, **i** Representative images of ovarioles without (EtOH control) or with methoprene treatment. G, germarium. Numerals indicate egg chamber stages. The stage 7/8 diapause arrest (asterisk in (**h**)) was released by methoprene treatment allowing the development of vitellogenic egg chambers (asterisk in (**i**)). **j**, **k** Arrowheads indicate GSCs. Dotted lines outline germarium regions 1 and 2, which are quantified in (**l**) (*p = 0.0265; **p = 0.0040). See also Supplementary Fig. 3. **m** Quantification of GSC numbers over time. **p = 0.0069; ***p = 0.0010; ****p < 0.0001. (*n*) number of germaria analyzed in (**l**, **m**). For **g**, **l**, **m**, data = mean ± s.e.m. Statistical analysis is by 2-way ANOVA before recovery and 1-way ANOVA with Tukey’s multiple comparisons test after recovery.

As diapause affected early stages of development, we asked whether GSCs were impacted. Therefore, we stained ovaries with anti-Vasa to mark germ cells and anti-Hu-li tai shao (Hts) to label spectrosomes (round organelles found in GSCs and daughter cystoblasts) and fusomes (elongated organelles found in young cysts) (Fig. 3a). GSCs are defined as Vasa^+^ cells, with an anterior spectrosome, that also contact the cap cells of the niche^34,35^. We compared the numbers of GSCs in germaria from flies aged at 18 or 25 °C (Supplementary Fig. 3a–d) to those in diapause and recovery (Fig. 3b–e). Quantification (Fig. 3f and Supplementary Fig. 3e) revealed that after 1 day at 10 °C, ovarioles contained the normal number of 2–3 GSCs (Fig. 3b, white arrowheads), indistinguishable from flies in non-diapause conditions. After 5 weeks at 10 °C, fewer Vasa^+^ cells with an anterior spectrosome were in contact with the niche (Fig. 3c, d, f). Some anterior, Vasa^+^ cells contained a fusome rather than a spectrosome (Fig. 3c, yellow arrowhead), or were in contact with the niche but contained a posterior spectrosome (Fig. 3d, yellow arrowheads). In contrast, germaria from post-diapause flies in recovery frequently contained two Vasa^+^ cells with an anterior spectrosome that were also in contact with the niche (Fig. 3e, Supplementary Fig. 3e).

GSC numbers declined over time in control flies maintained at 25 °C (Fig. 3f and Supplementary Fig. 3a–c)^35^. As expected, at 18 °C the decline was slower due to the known slower rate of ovarian development and aging at a lower temperature (Fig. 3f). If the ovarian response to diapause simply represented overall metabolic slowing due to the reduced rate of living of a cold-blooded animal, we would expect the rate of GSC loss to slow further at 10 °C compared to 18 °C. Instead, the rate of decline in GSC numbers in diapause more closely resembled the decline in non-diapausing flies at 25 °C (Fig. 3f, compare red and green lines) than 18 °C (Fig. 3f, compare green and blue lines), though the underlying cell biology differed (see below). Flies deprived of protein lost GSCs fastest (Fig. 3f, gray line).

We next counted GSCs in flies that had been in diapause for six weeks, then moved to recovery conditions. When control flies were aged for six weeks at 25 °C, then moved to vials with fresh yeast at 25 °C, a slight increase from 0.9 to 1.1 GSC per germarium occurred (Fig. 3f, red line at 42 and 44 days). After this slight improvement, likely due to the enriched food, GSC numbers resumed their decline over time. Strikingly, ovaries from flies that had been in diapause conditions for 6 weeks recovered from ~1 to ~2 GSCs per germarium, comparable to youthful GSC numbers (Fig. 3f, green line at 42 and 44 days). At 10 °C, GSC numbers were lower at all time points in long-day conditions compared to short-day (Supplementary Fig. 3f), demonstrating that both temperature and day length contribute to GSC preservation in diapause. Post-diapause GSCs also persisted longer than controls (Fig. 3f, 84 days). In contrast, >90% of protein-deprived flies died; the few survivors exhibited a transient increase in GSC number but died shortly thereafter (Fig. 3f, gray line). Together these data show that although many GSCs lose their defining morphological features during diapause, the potential to recover is maintained.

To further characterize the effects of diapause on GSCs, we used anti-phospho-histone H3 staining to measure the percentage of GSCs in mitosis. Two weeks of diapause caused a nearly threefold reduction in GSC mitoses compared to GSCs in flies maintained at 25 °C or at 18 °C (Fig. 3g). GSC mitotic activity declined faster at 25 °C than 18 °C over the next 2 weeks but remained stable in diapause. By 6 weeks, GSC mitotic activity declined further in all three conditions. Remarkably, when flies were moved to 25 °C after 6 weeks, GSC numbers rebounded better than those from flies kept at either 18 or 25 °C (Fig. 3g). These results show that diapause conditions are not purely stressful; rather, diapause preserves GSC mitotic potential that is lost in non-diapause conditions.

To determine which effects on early ovarian development were consequences of the established stage 7/8 yolk-uptake checkpoint and which effects were independent, we bypassed the checkpoint by treating diapausing flies with methoprene, a juvenile hormone (JH) analog that promotes yolk uptake. Diapausing flies produce less JH than non-diapausing flies, resulting in stage 7 arrest^8^ (Fig. 3h). Methoprene treatment significantly reversed developmental arrest and egg chamber degeneration, allowing development beyond stage 8 (Fig. 3i). Compared to vehicle-treated controls (Supplementary Fig. 4a–c), methoprene-treated ovaries doubled in size, and ~20% of ovarioles even produced mature eggs (Supplementary Fig. 4d–g). Interestingly, methoprene also depleted the germarium of cysts relative to the EtOH vehicle control (Fig. 3j, k), resulting in a marked and measurable reduction in the germarium area (Fig. 3l). This finding suggested that, during diapause, the stage 7 arrest caused early cysts to stall in their development.

In contrast, GSC numbers were indistinguishable between EtOH and methoprene-treated germaria in diapause conditions (Fig. 3m), indicating that the effect of diapause on GSCs was independent of JH. In post-diapause, methoprene-treated animals, GSC numbers did not recover quite as well as in EtOH controls (Fig. 3m), suggesting that arresting the development of cysts and egg chambers contributes to preserving GSC recovery potential during diapause. We conclude that diapause causes multiple, independent blockades that affect virtually all stages of egg chamber development.

### DNA damage, reactive oxygen species, and p53 in the GSC response to diapause

**Fig. 4:**
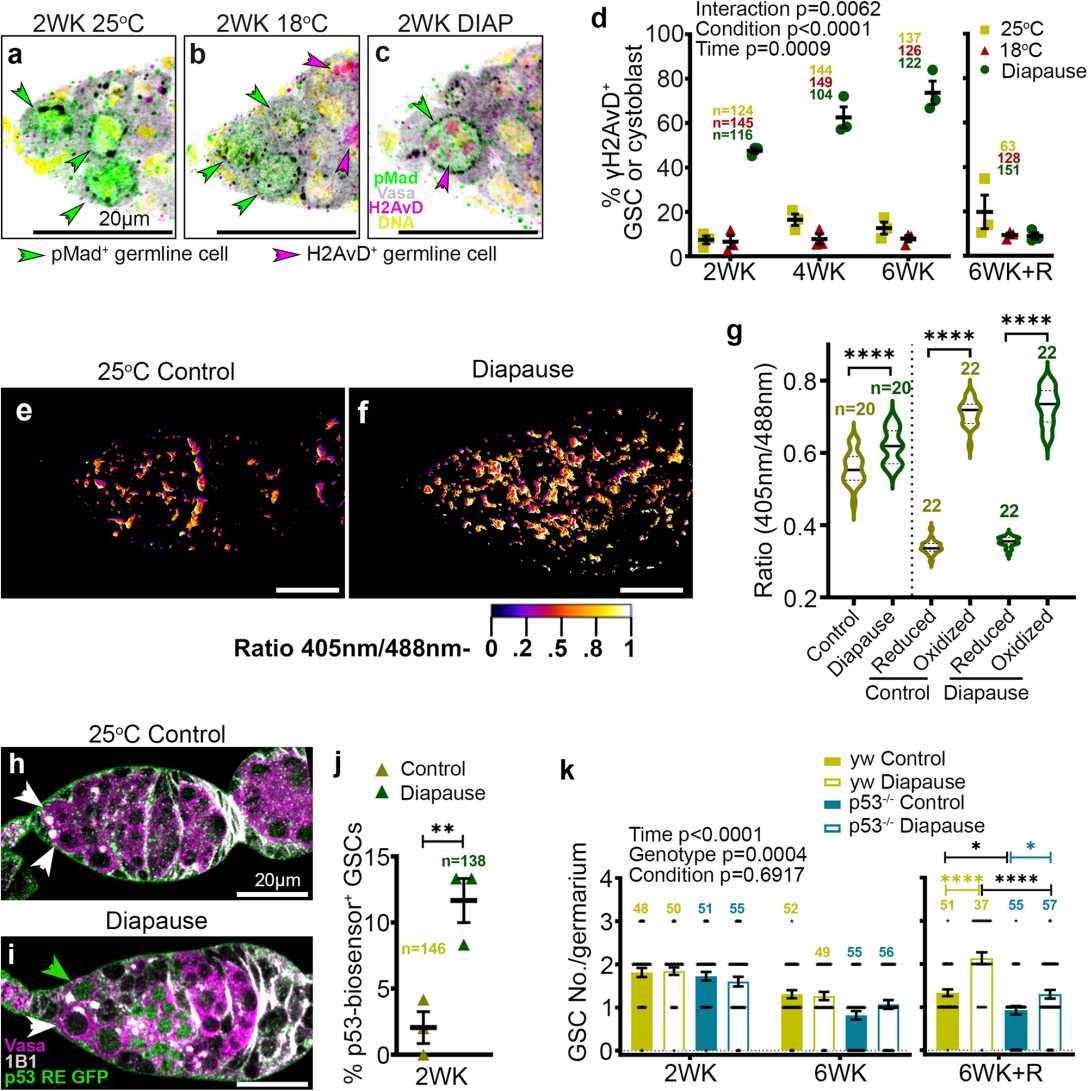
DNA damage, ROS, and p53 mediated protective response in diapause. **a**–**c** Confocal micrographs of germaria from flies maintained for 2 weeks (WK) at 25 °C (**a**), 18°C (**b**), or diapause conditions (**c**). Anti-pMad (green) labels cells with active Dpp signaling from the niche. Anti-γH2AvD (magenta) labels cells with double-strand DNA breaks due to damage [magenta arrowheads in (**c**)] or meiosis (arrowheads in (**b**)). **d** Percentage of H2AvD^+^ cells out of total pMad^+^ cells from 25 °C, 18 °C, and diapause over time. R, recovery. Statistical analysis is by 2-way ANOVA before recovery and 1-way ANOVA with Tukey’s multiple comparisons test after recovery. **e**, **f** Ratiometric (405 nm/488 nm) confocal micrographs of germaria from tubulin-mito-roGFP2-Orp1 flies kept for 2 weeks at 25 °C [control (**e**)] and in diapause (**f**). **g** Quantification of ROS in the germarium relative to maximally reduced (with dithiothreitol) or oxidized (with diamide, see methods for details). Statistical analysis is by 1-way ANOVA and Tukey’s multiple comparisons test. ****p < 0.0001. **h**, **i** Confocal micrographs of germaria from flies maintained for 2 weeks (WK) at 25 °C (**h**), or diapause conditions (**i**). Anti-Vasa labels germline cells (magenta) and 1B1 stains the Hts protein indicating spectrosome/fusome (GSCs are shown by arrowheads). p53 RE GFP-NLS biosensor (green) labels cells with p53 activity (green arrowhead in (**i**)). **j** Percentage of p53 RE GFP^+^ GSCs out of total GSCs from 25 °C control and diapause for 2WK. *p* value from unpaired two-tailed *t*-test. **p = 0.0095. **k** Quantification of GSC number/germarium from y, w control flies and p53^−/−^ in 25 °C control and diapause from 2WK, 6WK, and 6WK recovery (6WK + R). Statistical analysis is by three-way ANOVA before the recovery and one-way ANOVA after recovery. *n* is the number of GSCs analyzed in (**j**), in all other cases, *n* = number of germaria analyzed. Data are mean ± s.e.m. *p = 0.0211; *p = 0.0346; ****p < 0.0001. Scale bars are 20 µm.

Another stress that leads to GSC loss is DNA damage^36^. Therefore, we tested whether DNA damage accumulates during diapause using γH2AvD antibody staining. Little to no γH2AvD staining was evident in GSCs from controls maintained at 25 °C (Fig. 4a) or 18 °C (Fig. 4b). Strikingly, γH2AvD accumulated in the majority of GSCs during diapause (Fig. 4c, d). Moreover, this staining virtually disappeared during recovery (Fig. 4d).

Diapause can lead to metabolic alterations and stress in worms, insects, and mammals, which can cause accumulation of reactive oxygen species (ROS), leading to DNA damage. To determine if diapause conditions caused an increase in ROS, we used a mitochondrial ratiometric fluorescence ROS reporter^37^. In the reduced state the 405/488 nm emission ratio is low, and oxidation causes it to rise^37^. Compared to 25 °C controls (Fig. 4e), we found that germaria from flies in diapause conditions exhibited significantly higher ROS (Fig. 4f, g).

A common consequence of DNA damage is the activation of p53, so we used a GFP reporter of p53 transcriptional activity (p53 RE GFP)^38^ to assess the effect of diapause. Compared to control GSCs (Fig. 4h), which rarely exhibited GFP expression, GSCs in diapause (Fig. 4i) showed an approximately sixfold increase in p53 biosensor expression (Fig. 4j). Furthermore, GSC numbers failed to recover post-diapause in p53 mutant flies compared to controls (Fig. 4k). We conclude that p53 activation contributes to the preservation of GSC recovery potential in diapause.

### The Chk2 DNA damage checkpoint maintains GSC diapause arrest

**Fig. 5:**
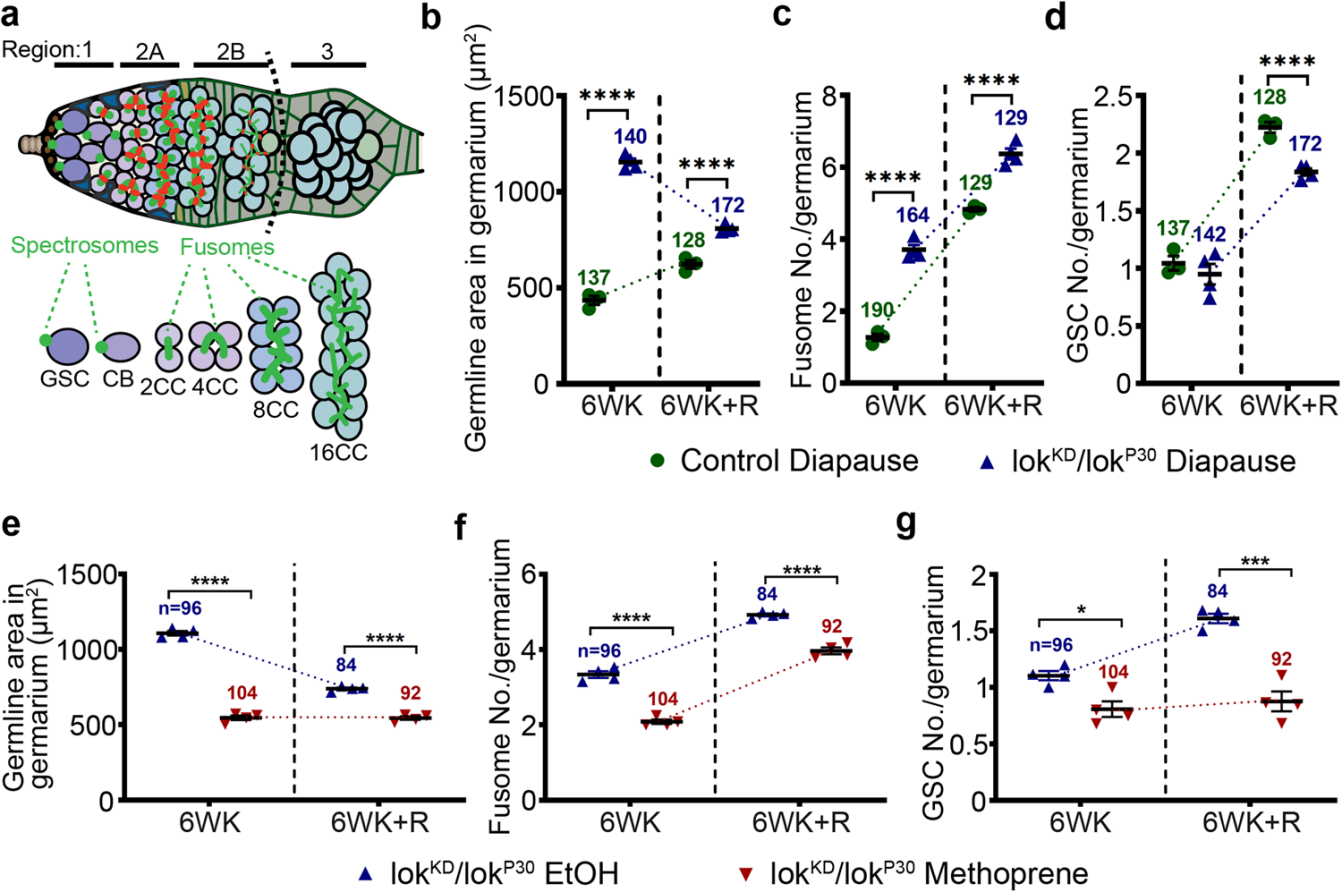
Chk2 DNA damage checkpoint maintains GSC diapause arrest. **a** Schematic of a germarium showing regions 1–3 and spectrosome/fusome morphology (GSC germline stem cell, CB cystoblast, CC cyst cell). Dotted line shows region 2/3 border. **b**–**d** Quantification of germline area in regions 1 and 2 (**b**), fusome number (**c**), and GSC number (**d**) in germaria from control and lok^KD/^lok^P30^. The control for **b**–**d** is Canton S. **e**–**g** Quantification of germline area in regions 1 and 2 (**e**), fusome number (**f**), and GSC number (**g**) in the germarium from lok^KD^/lokP^30^ treated with either ethanol vehicle (EtOH) or methoprene. Throughout Fig. 5, *n* = number of germaria analyzed. Data are mean ± s.e.m. from at least three independent experiments. Statistical analysis was carried out by unpaired two-tailed *t* test (*p = 0.0101; ***p = 0.0003; ****p < 0.0001). See also Supplementary Fig. 5.

The *loki* (*lok*) locus encodes *Drosophila* Chk2, which is a protein kinase that monitors genomic stress and leads to cell cycle arrest when defects are detected^39,40^. To test if Chk2 plays a role in the response to DNA damage incurred during diapause and/or in recovery post-diapause, we compared control and *lok* mutant germaria in diapause and recovery. Germaria are composed of three regions (Fig. 5a). Region 1 includes GSCs and their immediate daughters, the cystoblasts. Region 2 includes 2-, 4-, 8-, and 16-cell cysts. Sixteen-cell cysts become surrounded by follicle cells in Region 2B, and bud to form an egg chamber in Region 3.

*lok* mutant germaria filled with developing cysts, leading to a dramatic increase in the germline area relative to controls (Fig. 5b and Supplementary Fig. 5a–g). This result suggests that the Chk2-mediated DNA damage response normally blocks cyst development during diapause. Upon diapause exit, wild type (Supplementary Fig. 5d) and *lok* mutants (Supplementary Fig. 5h) exhibited similar germline areas (Fig. 5b), suggesting that Chk2 was neither active nor required post-diapause. Thus, during diapause, DNA damage accumulates and Chk2 blocks cyst development, whereas post-diapause the damage disappears, the block is released, and development resumes.

Fusomes are present between cells of developing 2-, 4-, 8-, and 16-cell cysts (Fig. 5a). In diapause, germaria from *lok* mutants contained more fusomes than wild type controls (Fig. 5c and Supplementary Fig. 5a’–g’). Post-diapause, fusome numbers rose in both wild type and *lok* mutant germaria (Fig. 5c and Supplementary Fig. 5d’, h’). Together these results suggest that Chk2 limits cyst development during normal aging, and even more significantly during diapause because of increased DNA damage (Supplementary Fig. 5i, j).

To determine whether the Chk2 developmental arrest is important for maintaining GSC potential during diapause, we compared GSC numbers between wild type and *lok* mutants during diapause and recovery. There was no measurable difference in GSC numbers between control and *lok* mutant germaria at 6 weeks of age in normal growth conditions (Fig. 5d and Supplementary Fig. 5k). However, the post-diapause rebound in GSC numbers was more limited in *lok* compared to wild type (Fig. 5d), indicating that Chk2-mediated developmental arrest contributes to the maintenance of GSC recovery potential. We conclude that GSCs incur DNA damage during diapause, which activates the Chk2 DNA damage checkpoint, reducing GSC division, and blocking cystoblast differentiation. Upon diapause exit, the damage is repaired and the checkpoint released, allowing development to proceed.

As both yolk deposition arrest and the early Chk2-mediated arrest contributed to GSC recovery post-diapause, we tested the effect of inhibiting both. Methoprene treatment of the Chk2 loss of function flies (lok^KD^/lok^P30^) for 6 weeks in diapause blocked the accumulation of cysts in the germarium (Fig. 5e, f and Supplementary Fig. 5l–m'). Furthermore, post-diapause recovery of GSCs was virtually abolished (Fig. 5g and Supplementary Fig. 5n–o'). We conclude that each arrest contributes independently to the maintenance of GSC potential.

### GSC–niche interactions in diapause and recovery

Niche signaling is of prime importance in stem cell maintenance. Therefore, we evaluated the cellular and molecular components of the niche during diapause and recovery. Cap cells form the GSC niche, and in non-diapause conditions there is an age-dependent decline in cap cell number^34,41^, which we confirmed (Supplementary Fig. 6a–c, g), using Traffic Jam (Tj) as a cap cell marker^42^. In diapause, cap cell numbers were similar to non-diapause over 4 weeks (Supplementary Fig. 6d, g). However, cap cell numbers were maintained slightly better in diapause by 6 weeks (Supplementary Fig. 6e, g), an effect that reached statistical significance by 10 weeks (Supplementary Fig. 6f, g).

**Fig. 6:**
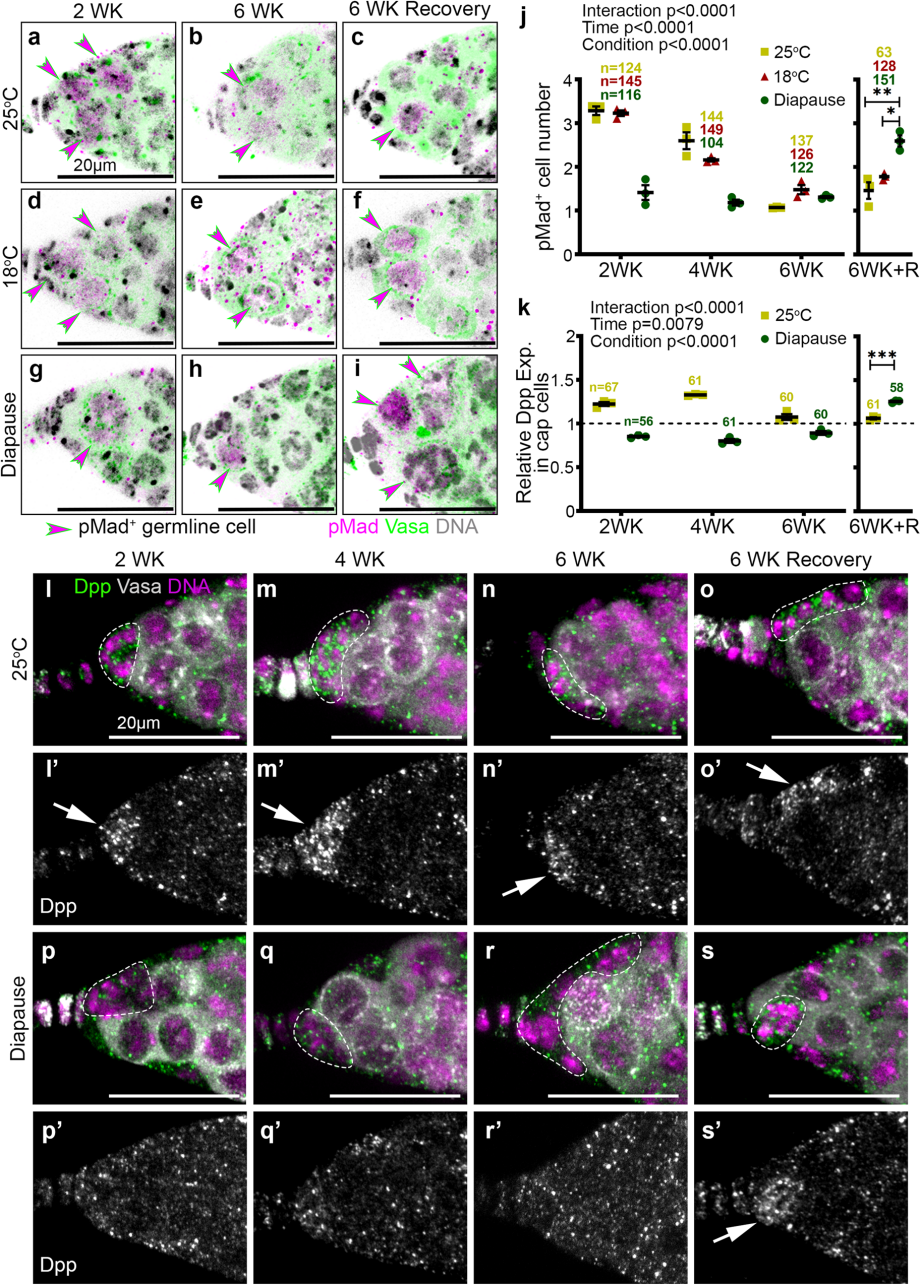
GSC niche signaling in diapause and recovery. **a**–**i** Confocal micrographs of germaria stained for pMad (magenta), Vasa (green), and Hoechst (gray) from flies maintained at 25 °C, 18 °C or in diapause. pMad^+^ cells are marked by magenta arrowheads. **j** Quantification of pMad^+^ germline cells over time from flies maintained at 25 °C, 18 °C, or in diapause. **k** Quantification of Dpp signaling in the cap cells relative to that in the germline region. **l**–**s** Confocal micrographs of germaria stained for Dpp (green) to identify the BMP signaling from the cap cells, along with Vasa (gray), and Hoechst (magenta) from flies maintained at 25 °C (**l**–**o**) or in diapause conditions (**p**–**s**) for 2, 4, 6, or 6 weeks (WK) followed by recovery. Cap cells are marked in dotted lines. (**l’**–**s’**) Dpp channel alone is in gray and Dpp enrichment in the cap cell region is shown with arrows. *n* = number of germaria analyzed. Data are mean ± s.e.m. from at least three independent experiments. Statistical analysis was carried out by two-way ANOVA before recovery time periods in (**j**, **k**). After recovery, ordinary 1-way ANOVA with Tukey’s multiple comparisons test for (**j**) and unpaired two-tailed *t*-test for (**k**) were undertaken (*p = 0.0119; **p = 0.0024; ***p = 0.0002). Scale bars are 20 µm. See also Supplementary Fig. 6.

Decapentaplegic (Dpp) produced by the cap cells is a key signal that maintains GSCs in the *Drosophila* ovary^43^. So, we compared niche signaling during diapause and normal aging using an antibody against phosphorylated Mothers Against Dpp (pMad), a reporter of Dpp signaling. We observed a decline in the number of pMad^+^ cells during normal aging at both 25 °C (Fig. 6a, b, j)^35,44,45^ and 18 °C (Fig. 6d, e, j). The reduction in pMad^+^ cell number was evident even earlier in diapause and remained low over time (Fig. 6g, h, j). A striking difference between 6-week-old control (Fig. 6c, f) and post-diapause (Fig. 6i) ovaries was the remarkable capacity to recover youthful numbers of pMad^+^ cells post-diapause (Fig. 6j).

To investigate the mechanism by which Dpp signaling was reduced during diapause and recovered post-diapause, we examined Dpp expression in the niche. As expected, Dpp was enriched in cap cells maintained for 2–4 weeks at 25 °C (Fig. 6k, l-m'); expression levels declined by 6 weeks (Fig. 6k, n, n') and did not improve under mock recovery conditions (Fig. 6k, o, o'). In contrast, we found little Dpp expression in the cap cells of germaria from diapausing conditions at any time point (Fig. 6k, p-r'). However, in post-diapause recovery, Dpp expression recovered to youthful levels (Fig. 6k, s, s'). We conclude that the recovery of Dpp signaling from the niche likely facilitates post-diapause recovery of GSCs.

### Post-diapause recovery of stalled GSC division

**Fig. 7:**
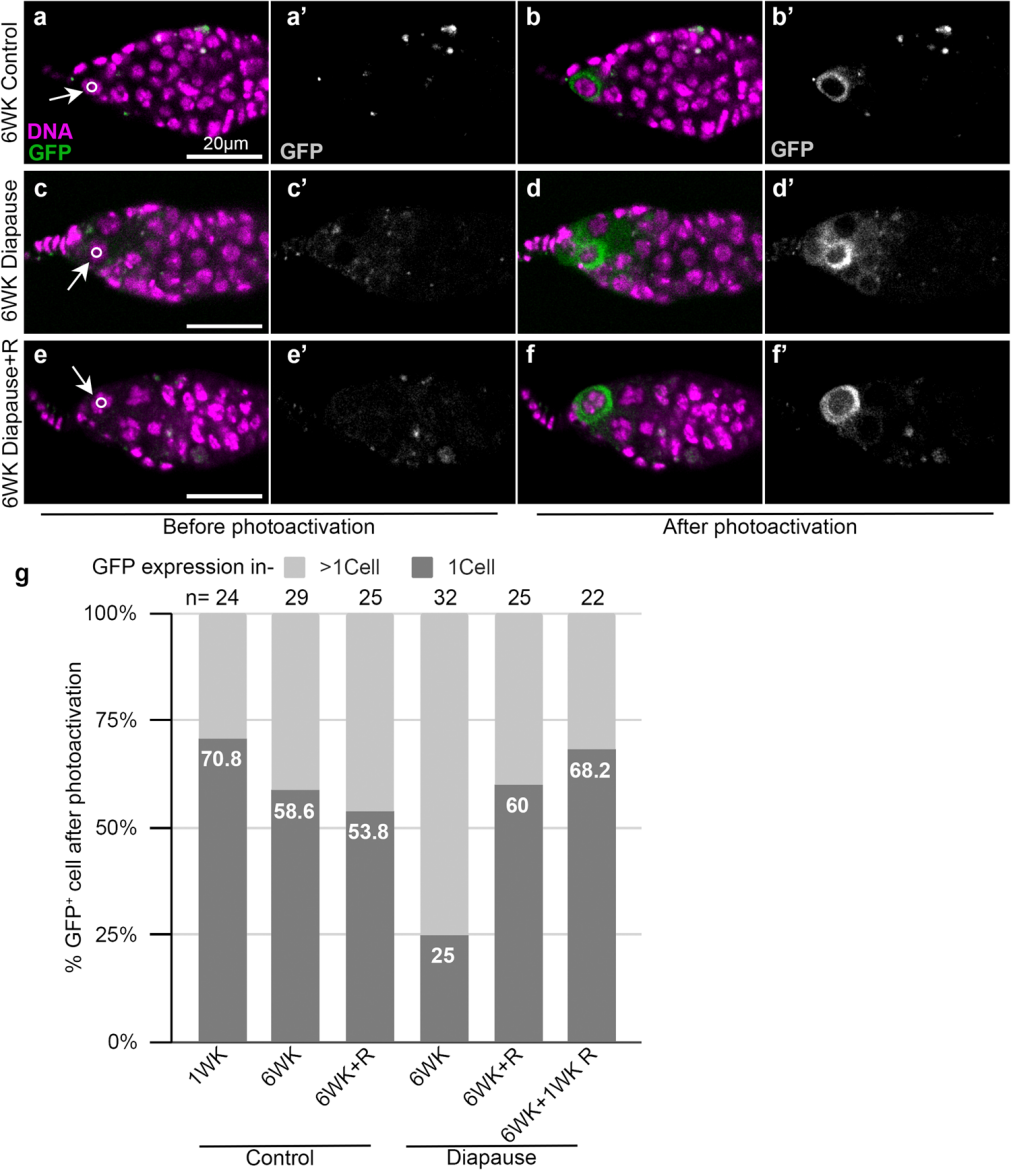
Incomplete cell division of GSCs in diapause. **a**–**f** Confocal micrographs of germaria from nosGAL4>UASp-PA(Photoactivatable)GFP-alphaTub84B flies kept at either 25 °C (**a**, **b** and **e**, **f**) or diapause (**c**, **d**) for the indicated time. Arrows indicate the anteriormost germline cell in each germarium and the circle shows the illuminated area. Images captured before (**a**, **c**, **e**) and after (**b**, **d**, **f**) photoactivation along with respective grayscale images are displayed (**a’**–**f’**). **g** Quantification of GFP confined to a single cell (1 cell) or to multiple cells (>1 cell). *n*, the numbers of photoactivation experiments carried out are shown above the bar. See also Supplementary Fig. 7.

During ovarian development under optimal growth conditions, GSCs divide asymmetrically to self-renew and produce a daughter cystoblast. To determine if cytokinesis was arrested, we expressed a photoactivatable GFP in the germline and illuminated the anteriormost germline cell with a 405 nm laser. If separation of the GSC from the cystoblast is complete, the GFP is retained in the single illuminated cell^46^. By contrast, if cells are connected, the fluorescent protein diffuses out of the illuminated cell into neighboring cells. In flies maintained in non-diapause conditions, the majority of GSCs retained the fluorescence in a single cell (Fig. 7a–b’, g). Even in wild type flies in optimal growth conditions, GSCs are known to remain connected to their daughter cystoblast by a ring canal for an extended period of the cell cycle^47^, and we found diffusion of activated GFP to >1 cell in ~30% of GSCs from 1-week-old flies, which increased to ~40% in 6-week-old flies. In diapausing flies, an even greater proportion GSCs (75%) remained connected to other cells (Fig. 7c–d’, g), a phenotype that reversed upon recovery (Fig. 7e–f’, g). We conclude that cytokinesis likely slows during normal aging, and the process slows even more in diapause. This phenotype reverses post-diapause, restoring GSC numbers.

Resolution of arrested cytokinesis appears to contribute to restoring GSCs with normal morphological characteristics post-diapause. Another mechanism that could theoretically contribute is the dedifferentiation of cystoblast or cyst cells back into GSCs, as has been previously observed following GSC ablation in both males^48^ and females^44^. During non-diapause ovarian development, GSCs divide to produce a daughter cystoblast, which in turn divides—this time with incomplete cytokinesis—to form a two-cell cyst. The two-cell cyst divides synchronously three more times, producing 4-, 8-, and eventually a 16-cell cyst. Ring canals form around the arrested cleavage furrows that connect cyst cells. dAnillin is a marker for ring canals and does not normally co-localize with Hts in GSCs or cystoblasts. However, in GSCs produced by dedifferentiation, dAnillin co-localizes with Hts on GSC spectrosomes^44^. Compared to controls (Supplementary Fig. 7a–c), we found dAnillin-positive spectrosomes in many (Supplementary Fig. 7e–g) though not all (Supplementary Fig. 7d) germaria recovering from diapause. These cells could be in the process of dedifferentiation though it is also possible that the dAnillin accumulates as a consequence of arrested cytokinesis. Similarly, cells with an anterior spectrosome that were also connected to a more posterior cell via a fusome may represent either cells arrested in cytokinesis or dedifferentiating precursors (Supplementary Fig. 7f, g and Supplementary Table. 1).

In addition to labeling ring canals, dAnillin is present in interphase nuclei of mitotically active germline and somatic cells (Supplementary Fig. 7a–n) and is lost from post-mitotic 16-cell germline cysts (Supplementary Fig. 7h–j) and from post-mitotic follicle cells (Supplementary Fig. 7a, stage 9)^49^. Compared to 25 °C controls (Supplementary Fig. 7i), fewer anterior germline cells exhibited nuclear dAnillin in diapause (Supplementary Fig. 7l, n). At 25 °C, cysts containing fusomes became enveloped by follicle cells and lost nuclear dAnillin and fusomes as they progressed out of region 2 (Supplementary Fig. 7h). However, during diapause, the fusomes and nuclear dAnillin staining disappeared from cells that remained within region 2 (Supplementary Fig. 7k, l, n), consistent with reduced mitotic activity. After 6 weeks in diapause, germline cells were mostly cystoblasts or 2-cell cysts. Four- and 8-cell cysts were rare, and 16-cell cysts with nuclear dAnillin and fusomes were essentially absent (Supplementary Fig. 7o). During diapause, dAnillin-negative and Hts-negative 16-cell cysts were present, though arrested (Supplementary Fig. 7l). Upon recovery, multicellular cysts reappeared along with GSCs (Supplementary Fig. 7m). These results suggest that the few remaining dAnillin-positive germ cells represent a pool from which GSCs may rebound post-diapause to produce developing cysts.

### Harnessing diapause mechanisms to slow normal GSC aging

**Fig. 8:**
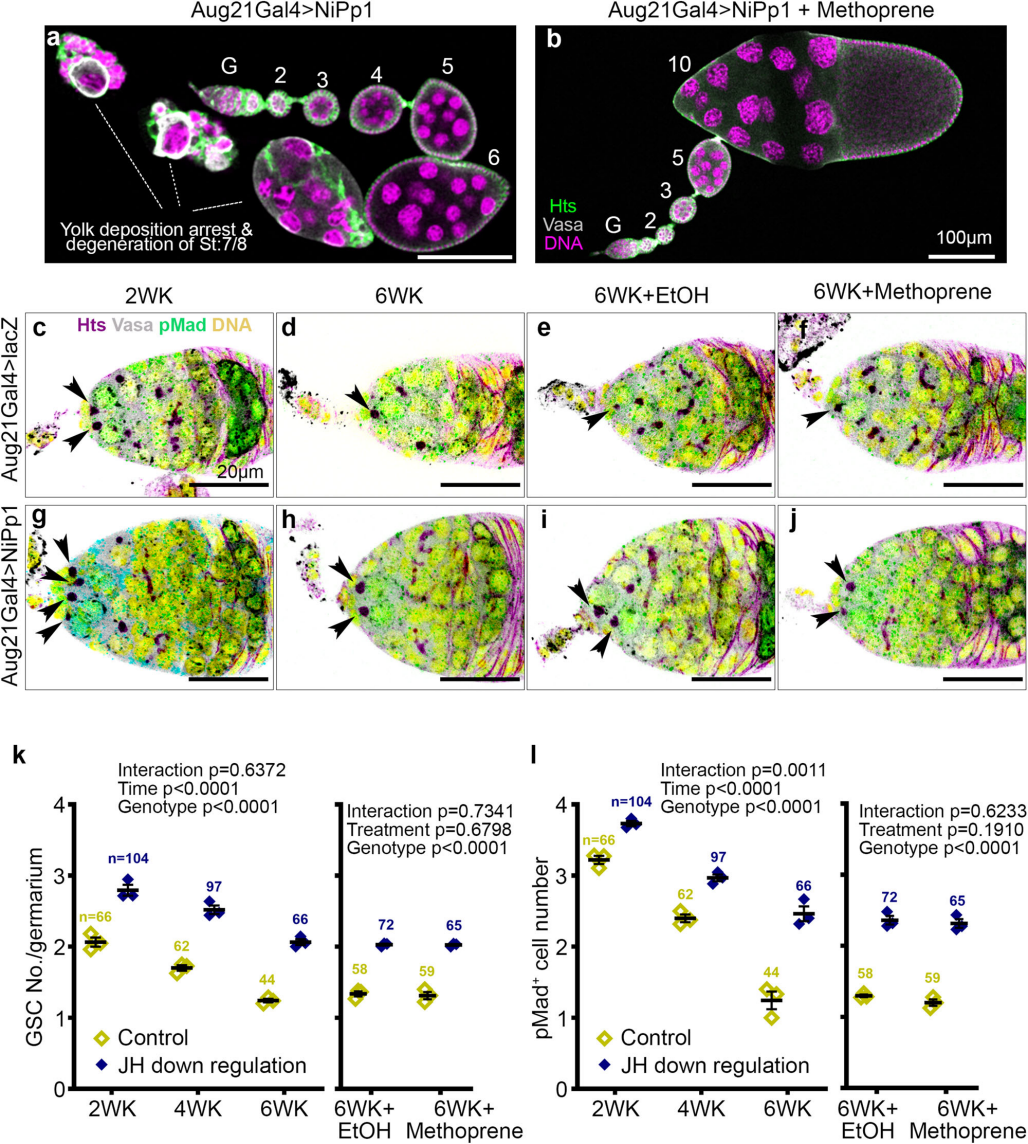
JH inhibition leads to a diapause-like arrest and GSC preservation in non-diapause conditions. **a** Confocal micrograph of ovarioles from a fly with Aug21Gal4 driving UAS-NiPp1 to inhibit JH production in the corpora allata. Egg chambers develop normally through stage 6, without early stage degeneration, then arrest at stage 7/8 (**a**), an arrest released by methoprene treatment (**b**). Scale bars are 100 μm (**a**, **b**). **c**–**j** Confocal micrographs of germaria from control (Aug21Gal4>UAS-lacZ, **c**–**f**) or JH downregulation (Aug21Gal4>UAS-NiPp1, **g**–**j**) stained for Hts (magenta), Vasa (gray), pMAD (green), and Hoechst (yellow) from flies maintained at 25 °C for 2 weeks (WK) (**c**, **g**), 6WK (**d**, **h**), or 6WK at 25 °C followed by supplementation with either EtOH (**e**, **i**) or methoprene (**f**, **j**). Arrowheads indicate GSCs and scale bars are 20 µm. **k** Quantification of GSC number over time from controls (Aug21Gal4>UAS-lacZ) and JH downregulation (Aug21Gal4>UAS-NiPp1) followed by methoprene treatment to mimic JH production in post-diapause recovery. **l** Quantification of pMAD^+^ cells over time in germaria from control flies and those with JH downregulation, followed by methoprene treatment. *n* = number of germaria analyzed. Data are mean ± s.e.m. from at least three independent experiments. Statistical analysis by two-way ANOVA was carried out separately before the treatment time periods and after the treatment. See also Supplementary Fig. 8.

Our results suggest that diapause arrest slows germline progenitor senescence and facilitates post diapause recovery, raising the possibility that mimicking the diapause state in flies maintained in optimal growth conditions might be sufficient to slow GSC aging. So, we inhibited JH production by expressing nuclear inhibitors of Protein phosphatase 1(NiPp1) using the corpora-allata-specific Gal4 (Aug21Gal4), which blocks vitellogenic development (Fig. 8a), as shown previously^50^, leading to small ovaries (Supplementary Fig. 8 compare c to a). Nearly 80% of the ovarioles showed stage 7 arrest. Control ovaries shrank and senesced over 6 weeks at 25 °C (Supplementary Fig. 8b), a phenotype that was not reversed by methoprene treatment (Supplementary Fig. 8e, f). Unlike normal aging, but similar to diapause, the ovarian effect of JH inhibition could be reversed by methoprene treatment (Fig. 8b and Supplementary Fig. 8a–h). Therefore, inhibition of JH production mimics diapause stage 7 arrest.

Comparing control flies expressing lacZ in the corpora allata (Fig. 8c–f) to those expressing NiPp1 to inhibit JH production (Fig. 8g–j), revealed JH inhibition resulted in a significant increase in GSC numbers (Fig. 8k) and Dpp signaling (Fig. 8l), better even than in flies maintained at 18 °C (Fig. 3f). We conclude that JH contributes to GSC loss and ovarian senescence during normal aging, and reducing JH production captures some of the GSC longevity preserved in diapausing flies.

## Discussion

The results of this study provide insight into the cellular and molecular mechanisms that drive diapause arrest of ovarian development and facilitate recovery. It has long been appreciated that *Drosophila* ovarian development arrests at stage 7/8 during diapause. However, effects on earlier stages, especially GSCs and early progenitors, had not been reported, and recovery mechanisms were unknown. While the yolk-uptake checkpoint is common to multiple stressful conditions, this work shows that diapause causes a more complete arrest compared to stresses like protein deprivation, predator exposure, and heat stress. We found that during diapause, the yolk uptake checkpoint also prevents earlier stage egg chambers from progressing through development, so they slowly degenerate. The germarium also fills with arrested cysts. When treated with methoprene, which releases the checkpoint, egg chamber development resumes, and the germarium empties out. However, activating the checkpoint by inhibiting the corpora allata from producing JH was not sufficient to arrest early-stage development. Therefore, it is the combination of the stage 7/8 arrest and either low temperature or some other feature of the diapause program that blocks the development of younger egg chambers. We conclude that different stresses produce overlapping but distinct responses.

**Fig. 9:**
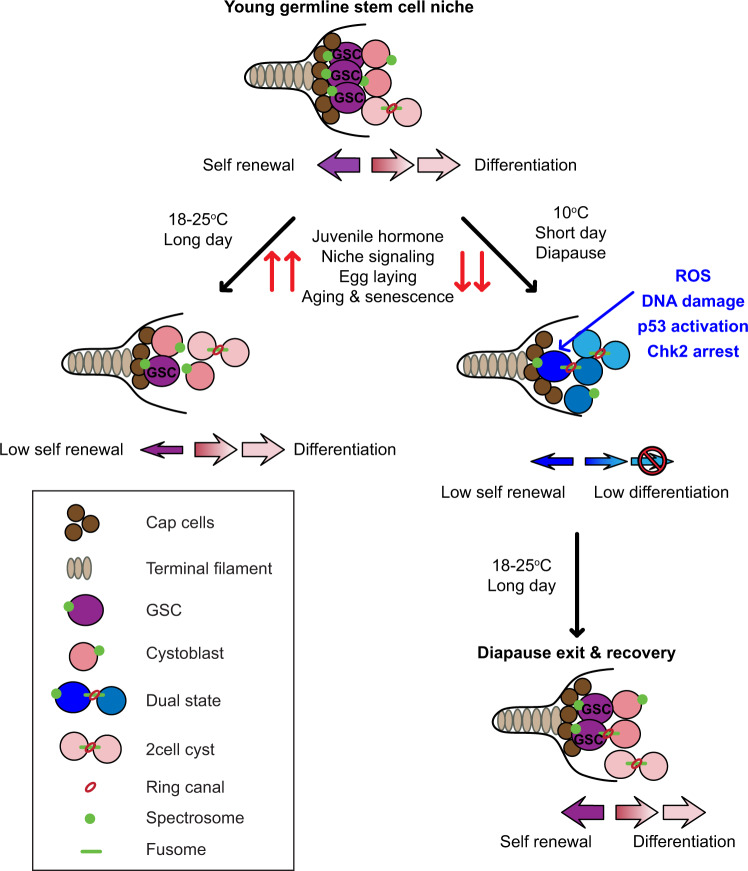
Model for ovarian diapause arrest and recovery. During diapause, the GSCs enter a state of inactivity mediated by Dpp downregulation, increased incidence of ROS, DNA damage, and Chk2 activation. The low level of differentiation maintains a pool of germline cells within the germarium, which replenishes GSCs upon diapause exit. Post-diapause, Dpp signaling from cap cells is restored and re-activates GSCs to reverse the developmental arrest.

Consistent with this theme, diapause causes profound impacts on GSCs (Fig. 9) that are independent of the stage 7/8 checkpoint. Niche signaling, which normally promotes GSC division and self-renewal, is reduced, cytokinesis slows, and cyst development is blocked, the combination of which arrests progenitor cells in an atypical state in which they neither self-renew nor differentiate. Flies and GSCs are more resilient to diapause conditions than to protein deprivation and heat stress because most protein-deprived and heat-stressed flies perish, and even those that survive do not recover. In contrast, post-diapause flies have more GSCs than flies maintained in maximal growth conditions and are able to maintain them for an additional 6 weeks.

We were surprised to find that GSCs are not maintained in a stem cell state during diapause, even though ovaries can rebound post-diapause to youthful stem cell numbers, proliferation, and ovary size. This remarkable recovery potential appears to result from a combination of mechanisms. JH levels are reduced during diapause, and mimicking that condition is sufficient to enhance GSC longevity in non-diapausing flies. However, JH reduction does not fully mimic diapause. Rather, each of the developmental blockades appears to provide some benefit. The persistence of slowly degenerating egg chambers appears to contribute to preserving reproductive potential, perhaps by reducing the normal drive to generate more egg chambers. In addition, reduced mitoses in the germarium may preserve the proliferative potential of germline precursor cells. Concomitant reductions in mitoses and differentiation appear to result from ROS and DNA damage, which activates Chk2 and p53. Germ cell progenitors thus become arrested, mostly in connected pairs of a GSC and a cystoblast (Fig. 9). When animals return to optimal growth conditions, the cell cycle resumes, as do robust self-renewal and differentiation programs. Thus, completion of cytokinesis and resumption of the cell cycle likely replenish the GSC pool in post-diapause recovery.

Two other mechanisms have been reported to replenish depleted GSCs: dedifferentiation of cystoblasts and symmetric division of remaining GSCs. The colocalization of dAnillin and Hts in some post-diapause GSCs supports the idea that dedifferentiation could play a role, as does the observation that depleting germaria of most cells but not GSCs (diapause + Methoprene, Fig. 3k) impairs post-diapause recovery. The possibility that symmetric GSC division also contributes to GSC recovery has not been tested.

It is interesting that the presence of degenerating egg chambers and DNA damage somewhat paradoxically preserve the reproductive potential of early germline progenitor cells. In many examples of stress responses, mild or transient stress can enhance cellular and organismal survival even when more extreme exposures cause death. As stem cell exhaustion likely contributes to aging and age-related diseases, further insights into the stress responses that preserve rather than eliminate stem cells may be key to anti-aging treatments.

## Methods

### Ethical Statement

*Drosophila* and wasp strains were obtained and reared according to standard protocols and institutional regulations from the University of California Santa Barbara.

### *Drosophila* stocks and culturing

For the majority of our experiments, we used Canton-S (CS) obtained from Bloomington *Drosophila* Stock Center (BDSC #64349). Stocks were amplified at 25 °C in food collection bottles with 15 female flies and 10 male flies and flipped every 3rd day. Virgin collections were carried out making sure no flies cross 6 h of age. We also generated heat shock-induced GFP mosaics using the FLP/FRT system by using flies obtained from BDSC-Ubi-FRT-STOP-FRT-nEGFP (BDSC #32251) and hs-FLP (BDSC #55815). Heat shock was carried out on a 37 °C water bath for 10 min and shifted immediately to their respective temperature as mentioned in the experiments. lok^KD^ and lok^P30^ alleles were a kind gift from Pamela Geyer. lok^P30^ is an imprecise excision of the P{EPgy2}EY15840 insertion, resulting in a deletion of the 5′ UTR, and the first two coding exons of lok and lok^KD^ is the D286A amino acid replacement (responsible for CHK2 kinase activity) resulting in a kinase-dead lok^36^. The yolk deposition arrest at normal development was carried out by using stocks Aug21-GAL4 (#30137) and UAS-HA-NiPp1 (#23711) obtained from BDSC^51^. Other fly lines used are P53R-GFP NLS biosensor (kind gift from John M Abrams), p53[5A-1-4] (BDSC #6815), Bam^Δ86^ (BDSC #5427) and bam deficiency (BDSC #27401).

### Environmental stress induction

All the diapause experiments are carried out in a cold room with 10 °C temperature and relative humidity of 40% in order to avoid fluctuation of temperature/conditions by incubator door openings. To assess the effects of diapause on ovary development, we subjected less than 6 h old virgin CS females to either 10 °C or 8 L:16D photoperiods (diapause conditions), 18 or 25 °C at 12L:12D photoperiod for 6 weeks. We then allowed the flies to recover by transfering them into vials with fresh food dusted with dry yeast for 1 day at 25 °C. To avoid sudden drastic change to the diapausing flies, we moved the flies from diapause to 18 °C for the first day and then to 25 °C. Their recovery status was maintained thereafter by transfering them every other day into a fresh fly food vial without dry yeast powder. Flies kept at 25 °C prior to recovery were transfered every other day while flies kept at 10 and 18 °C were only transfered for recovery and beyond. To assay the effects of nutrient deprivation on ovary development, we collected virgin CS females and placed them in empty vials containing a Kimwipe soaked with a 5% (v/v) molasses/water solution^52^. The solution was made daily and the flies were transfered daily to prevent fungal growth in the molasses solution. After 6 weeks of deprivation, the flies were transfered to fresh vials containing standard fly food medium with dry yeast for a day for the recovery. The death rate was high in protein deprivation and more than 90% of flies died within.

Heat stress experiments were carried out at 30 and 33 °C in incubators with LED light set to a 12L:12D cycle. A long day (16L:8D) experiment at 10 °C was carried out using an LED light, to avoid heat emission, in the 10 °C room.

### Immunostaining and antibodies used

Immunofluorescence was carried out using standard procedures. Briefly, ovarioles were dissected in phosphate-buffered saline (PBS) using a bent tungsten needle by pulling on the stalk region of older egg chambers to minimize damage to the germarium. Ovarioles that were prepared for immunostaining were fixed in 4% paraformaldehyde (PFA) in PBS for 20 min. Fixing for dAnillin staining was carried out for 10 min in PBS containing 3.7% formaldehyde. To avoid sticking of samples, 20 µl of PBST (PBS + 0.2% Triton X100) was added to the fix solution. After fixation, ovarioles were washed 3 times for 10 min each in PBST and then incubated with primary antibodies at 4 °C overnight. The following morning, the ovarioles were washed twice for 15 min in PBST prior to incubation with secondary antibodies for at least 2 h at room temperature. After removal of secondary antibodies, the samples were washed 3 times in PBST for 10 min each. Hoechst was used as a nuclear stain and was added to the second wash solution. The ovarioles were subsequently mounted in vectashield and stored at 4 °C until imaged. All dilutions were made into PBST. The small diapause ovaries required an extra 5 min to settle before removing solutions from the tube at each step. We followed the same procedure for all controls as well. The antibodies used in this study are Vasa, Hts(1B1), γH2AvD (DSHB); Tj (kind gift from Dorothea Godt); pH3(Cell Signaling Tech); Smad (Abcam, ab52903); Dpp (R&D SYSTEMS, MAB159) and dAnillin (kind gift from Christine Field).

### Imaging and processing

All imaging was carried out on a Zeiss LSM-780 confocal microscope, using 63× oil immersion objective and a 20× as a reference to the ovariole. The whole ovary images were taken on a Zeiss axio zoom microscope without any staining. Representative images were processed in Fiji and exported as TIF files. The exported images were cropped and all figure panels were made in Adobe Photoshop CC. The images detecting  cap cells, DNA damage, and dAnillin staining were inverted in Photoshop for better contrast. Only the z-sections that included the cells of interest were projected for each image to avoid multiple unwanted cells obscuring them. Most of the visualization and quantifications were carried out using Imaris and/or Fiji software.

### GSC quantification

GSCs are characterized as the anterior-most vasa-positive cells in the stem cell niche that display an hts signal(spectrosome) anteriorly. The GSCs were counted in Imaris software by comparing both 3D images and z-slices individually.

### JH analog treatment

In order to investigate the effects of JH on ovary development during diapause, we first placed virgin CS females less than 6 h old in diapause conditions. To each vial, we then inserted a cotton swab saturated with either 10 μl of 200 μg/ml of methoprene pestanal (from Sigma #33375) in ethanol or only 10 μl of ethanol for vehicle control. This concentration was 10 times more than required to completely rescue the yolk deposition arrest at normal conditions^50^. The cotton swab was supplemented with methoprene at the same concentration on every other day.

### Area quantification

The germline area was calculated using the Vasa channel on total intensity projected (sum slices) images by marking the region up to region:2 in the germarium. The area with in the marked region was limited to the auto threshold. Both the control and experiments were taken using the same conditions and microscope settings.

### Cap cell quantification

Cap cells were identified using the Traffic jam (Tj) antibody which was a kind gift from Dorothea Godt. Cap cells were differentiated from escort cells easily as they were round and stained less intensely for Tj. All counting was performed using Imaris software.

### Dpp quantification

The relative Dpp signaling from the cap cells was measured as background-subtracted mean intensity by marking the cap cell area in a total intensity projected image and normalizing it to that of the same area in the germline cell region.

### pMAD and H2AvD quantification

The pMAD positive cells were detected by visualizing in 3D and 2D using Imaris software and manually counting them at the anterior-most within the germarium. γH2AvD positive cells were detected, counted manually in these pMAD positive cells by comparing the level of γH2AvD in all pMAD positive cells and neighboring pMAD negative cells.

### Statistics and reproducibility

All statistical analyses and graphs were made using the software SigmaPlot or GraphPad Prism. A minimum of three independent experiments were conducted and shown as data points in graphs. Total germaria/samples analyzed are shown on top of each graph. The statistical tests used are described in the figure legends. An unpaired *t*-test was used to compare two groups and an ordinary one-way ANOVA along with Tukey’s multiple comparisons test for multiple groups. Whenever we followed experiments for different time periods before recovery, we have used either two-way ANOVA or three-way ANOVA (in Fig. 4p). All of the representative images are chosen to depict the majority/average value from the quantifications.

### Ratiometric ROS assay and analysis

ROS status of the germarium in diapause and control was carried out using the ratio-metric mitochondrial ROS sensor—tub-mito-roGFP2-Orp1 (BDSC #67673). The flies were kept at diapause or 25 °C and dissected in PBS containing 20 mM N-ethylmaleimide (NEM, sigma #E3876) to conserve the redox state of roGFP2. Samples were fixed with 4% PFA for 15 min at room temperature. PFA was washed off in PBS twice for 10 min each and samples were equilibrated in 80% glycerol containing 0.4% N-propyl gallate (sigma #02370) in PBS overnight. Dynamic range (DR) between fully reduced to fully oxidized germarium or GSC region and the difference between 25 °C and diapause conditions were measured using exogenous addition of a reductant, 10 mM DTT (dithiothreitol, sigma # D9163) for 5 min or an oxidant, 1 mM DA (diamide, sigma #D3648) for 2 min before being blocked with NEM.

Images were captured on Zeiss LSM780 with the same setting for all the samples and processed using ImageJ. Briefly, the background was subtracted using the rolling ball radius set to 50 pixels. Pictures were then converted to 32-bit format. The intensities of the 488 nm images were thresholded, and values below the threshold were set to “not a number” (NaN). A ratiometric image was created using the “Ratio plus” plugin by dividing the 405 nm image by the 488 nm image and displayed using the lookup table “Fire.” The analysis was carried out on the whole germarium and also the anterior-most region of the germarium where GSCs are found.

### Photo-activation experiment

For photo-activation experiments, UASp-PAGFP-alphaTub84B (#32075) stock crossed to *nos*-GAL4 vp16 (kind gift from Jecelyn McDonald) was used. Flies kept at either 25 °C or diapause were dissected, washed, and mounted in Schneider’s media with Hoechst (2 µg/ml) on a lumox dish. Photo-activation was carried out on the anterior-most germline cell using a 405 nm laser line with a power set at 15 and opening the pinhole to the maximum on a 40× oil immersion objective for 3 cycles. Images were captured before and after the photo-activation.

### Reporting summary

Further information on research design is available in the Nature Research Reporting Summary linked to this article.

## Supplementary information

Supplementary Information

Reporting Summary

## Data Availability

Complete data are available in the main article, supplementary materials, and source data files. Since all the raw confocal imaging data supporting the findings of this study runs around two terabytes and in multiple files, we have not submitted it to the public repository but preserved in our NAS drive and are freely available from the corresponding author or the first author (Contact Address—dmontell@ucsb.edu; easwaran@ucsb.edu. Representative images are in the main or supplementary figures. Source data are provided with this paper.

## Source data

Source Data
