# Supplementary material for: Enhanced germline stem cell longevity in Drosophila diapause
Source: Nat Commun. 2022 Feb 7;13:711. doi: 10.1038/s41467-022-28347-z (PMC8821637; doi:10.1038/s41467-022-28347-z)
Supplement: Supplementary file 1 — Supplementary Information [file 41467_2022_28347_MOESM1_ESM.pdf]

Enhanced germline stem cell longevity in *Drosophila* diapause

Sreesankar Easwaran[1], Matthew Van Ligten[1], Mackenzie Kui[1], and Denise J. Montell[1]*

[1]Molecular, Cellular, and Developmental Biology Department

University of California

Santa Barbara, CA 93106

*author for correspondence

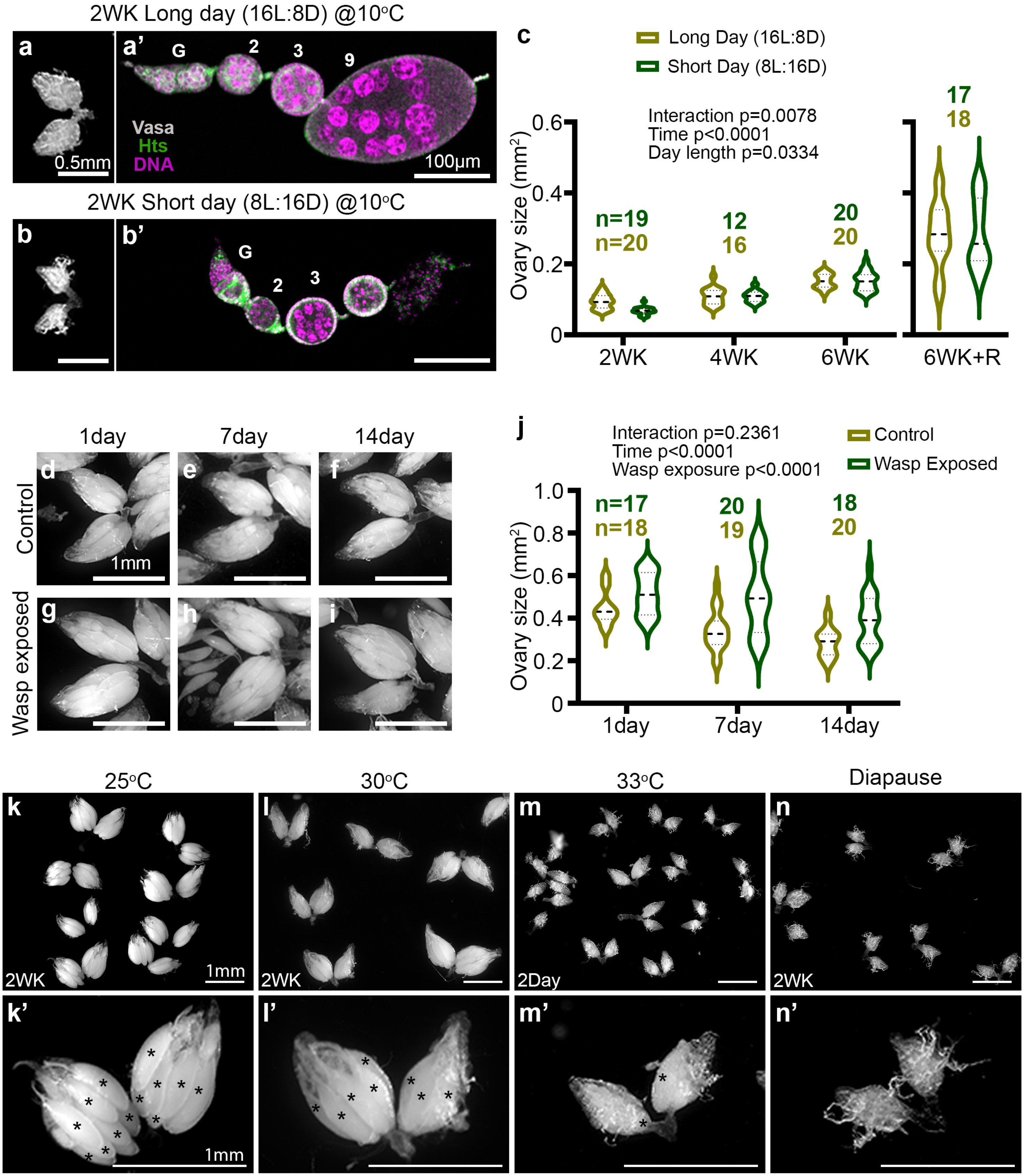

2WK Long day (16L:8D) @10°C
a
a'
G
2
3
9
Vasa
Hts
DNA
0.5mm
100μm
2WK Short day (8L:16D) @10°C
b
b'
G
2
3
c
Long Day (16L:8D)
Short Day (8L:16D)
Interaction p=0.0078
Time p<0.0001
Day length p=0.0334
Ovary size (mm²)
n=19
n=20
12
16
20
20
17
18
2WK
4WK
6WK
6WK+R
1day
7day
14day
Control
Wasp exposed
d
e
f
g
h
i
1mm
j
Interaction p=0.2361
Time p<0.0001
Wasp exposure p<0.0001
Control
Wasp Exposed
n=17
n=18
20
19
18
20
25°C
30°C
33°C
Diapause
k
l
m
n
2WK
2WK
2Day
2WK
1mm
k'
l'
m'
n'

Supplementary Fig. 1: Effect of different environmental conditions on ovary development.

a-b Darkfield micrographs of whole ovaries from long day (**a**) and short day (**b**) at 10°C along with respresentative ovarioles (**a'&b'**) at higher magnification. **c** Violin plots showing quantification of ovary size. Statistical analysis is by 2way ANOVA before recovery and unpaired two-tailed t test after recovery. **d-i** Darkfield micrographs of whole ovaries from unexposed control flies (**d-f**) or flies exposed to predatory wasps over time. **j** Violin plots showing quantification of ovary size in wasp-exposed compared to unexposed flies. Statistical analysis is by 2way ANOVA. **k-n** Darkfield micrographs of whole ovaries from flies kept at the indicated temperatures for 2 weeks (**k-l**) or 2 days (**m**) compared to the diapause for 2 weeks (**n**). **k'-n'** Higher magnification of a pair of ovary from **k-n**. Asterisks indicate mature eggs. Numbers of ovaries analyzed (n) are shown above the plots in **c** and **j**.

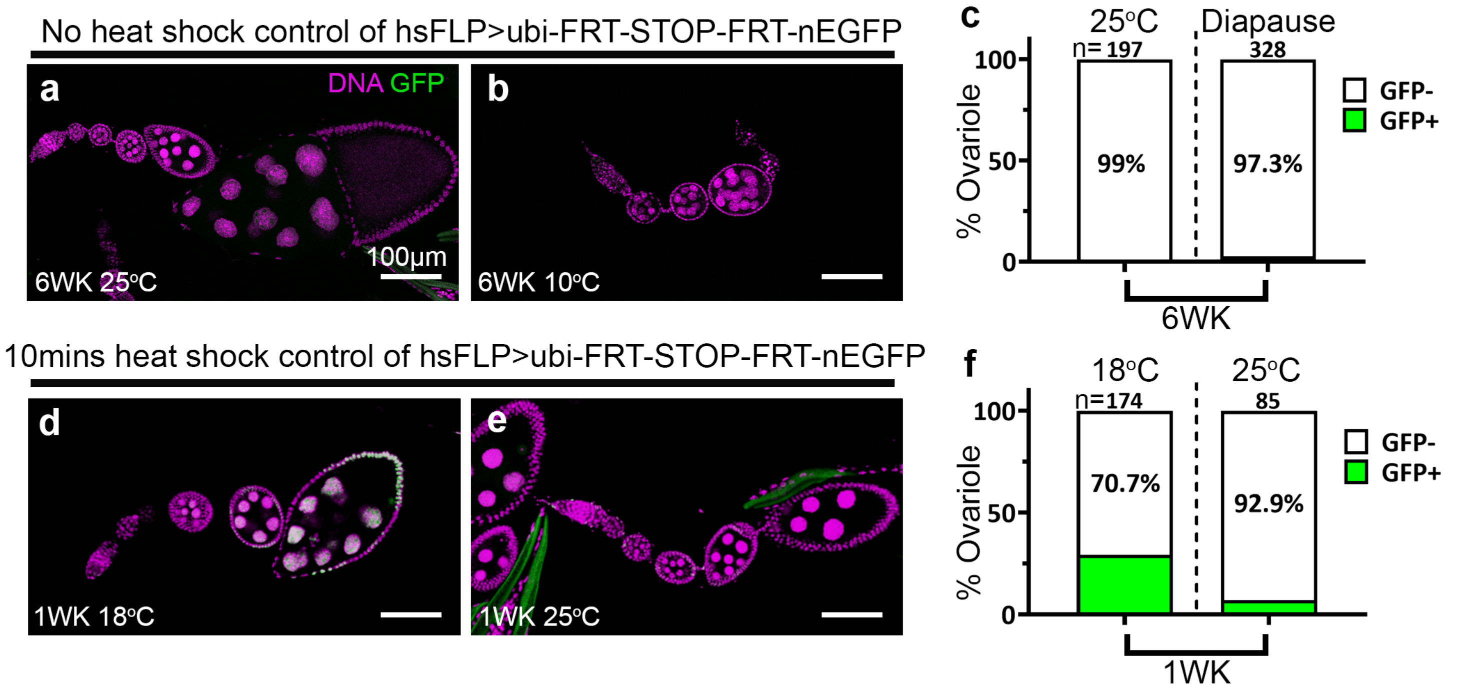

Supplementary Fig. 2: Multistage diapause arrest.

a-c No heat shock controls after 6 weeks at either 25°C (**a**) or diapause (**b**) and quantified in **c**.

d-f 10 minutes heat shock followed by 1 week at 18°C (**d**) or 25°C (**e**) and quantified in **f**. Scale bars are 100 µm. n, numbers of ovarioles analyzed.

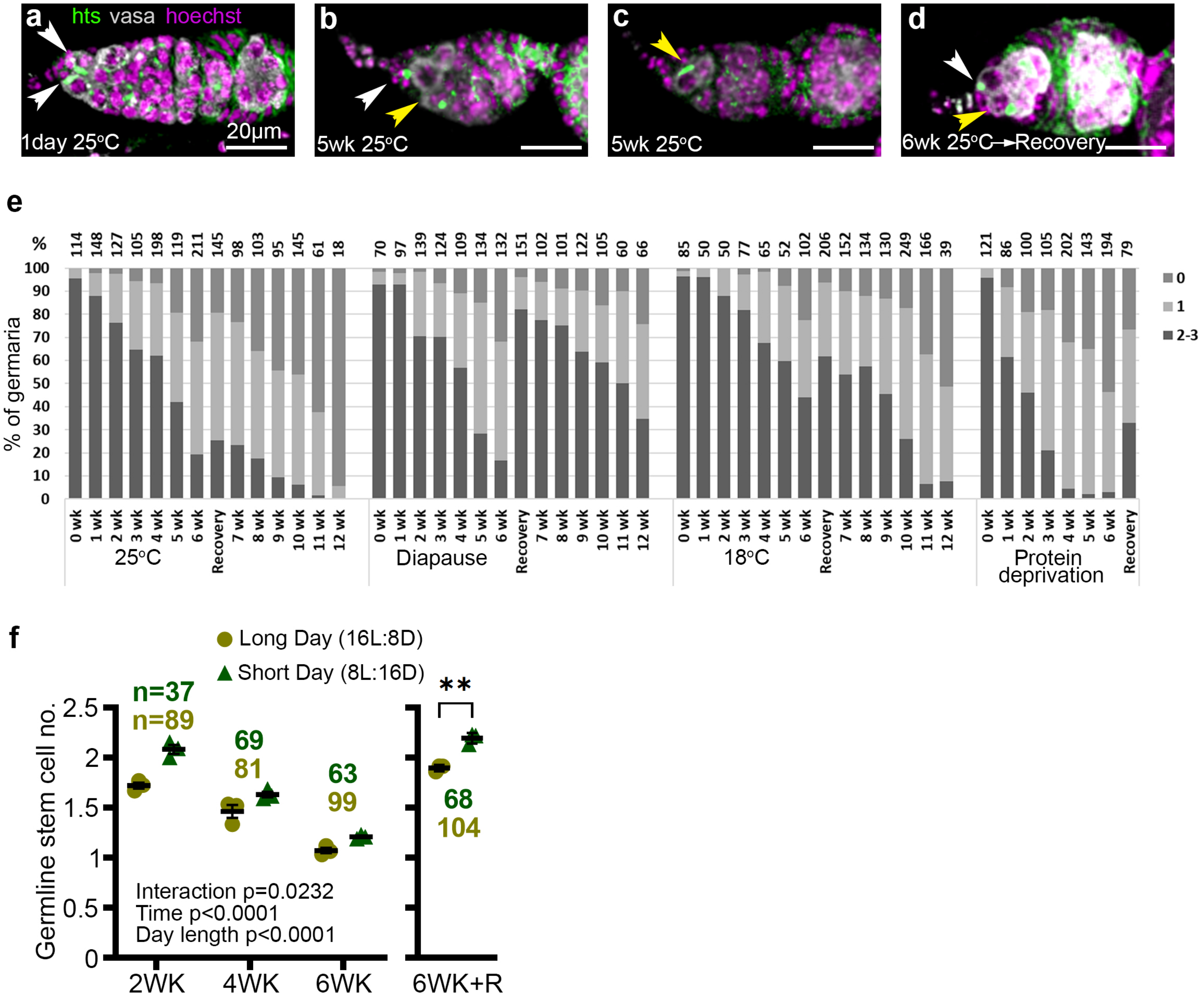

Supplementary Fig. 3: Germline stem cells in diapause and recovery.

a-d Confocal micrographs of germaria from flies aged at 25°C. Arrowheads depict GSCs (white) or cystoblasts (yellow). Scale bars are 20μm. **e** Percentage of germaria with the indicated numbers of GSCs plotted from the same data as shown in Fig. **3f**. **f** Germline Stem Cell (GSC) quantification from the ovarioles dissected from flies kept at 10°C at different day lengths. Data are mean ± s.e.m. from at least 3 independent experiments. n, numbers of germaria analyzed are shown above the graphs. 2way ANOVA was carried out before recovery and unpaired two-tailed t test after recovery. **, p=0.0012.

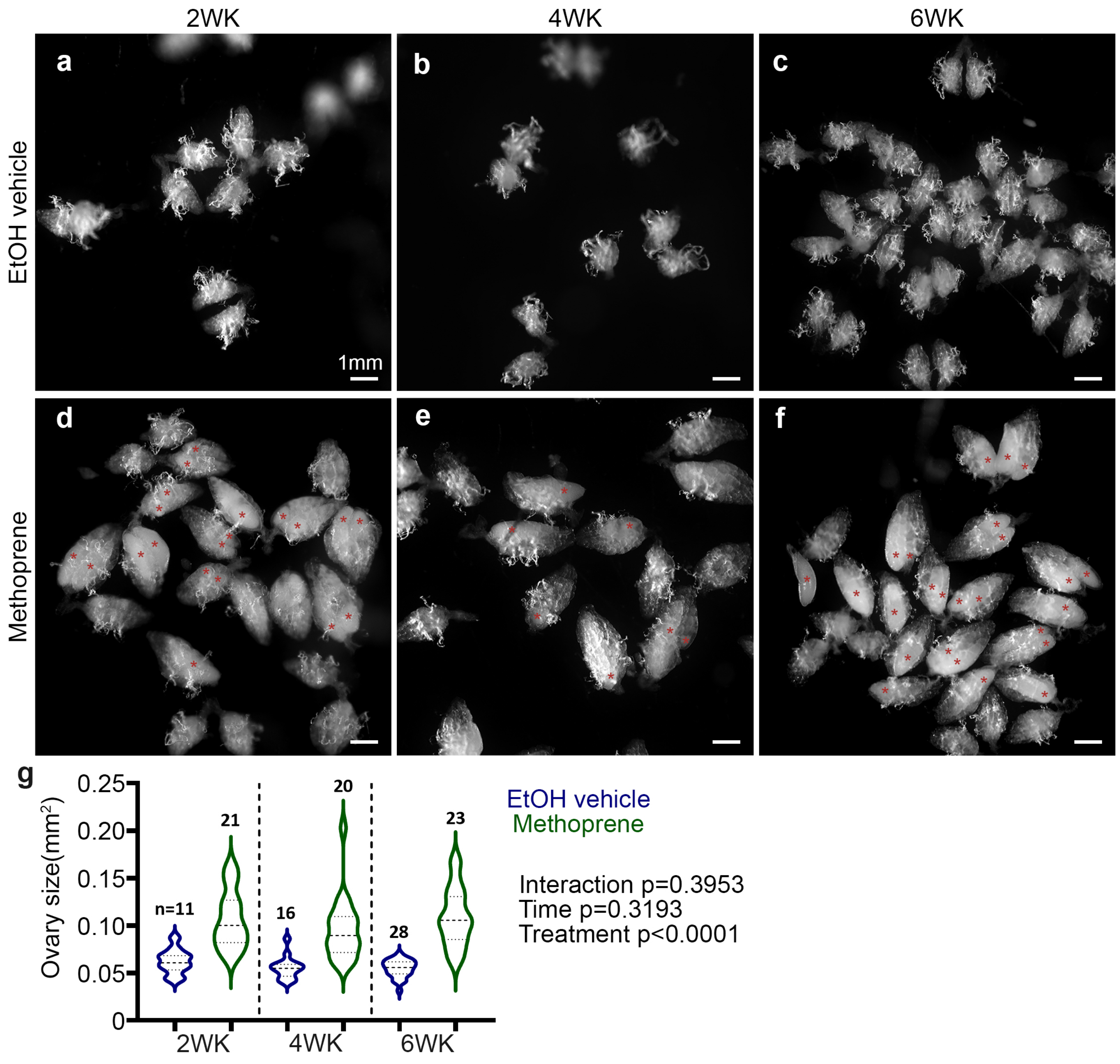

Supplementary Fig. 4: Release of yolk deposition checkpoint by methoprene treatment.

a-f Darkfield micrographs of whole ovaries from flies treated with EtOH (vehicle control) or methoprene for the indicated times. Scale bars are 1mm. Asterisks label egg chambers ≥ stage 12. **g** Quantification of ovary size. Numbers of ovaries analyzed are shown above. Statistical analysis was carried out by using 2way ANOVA.

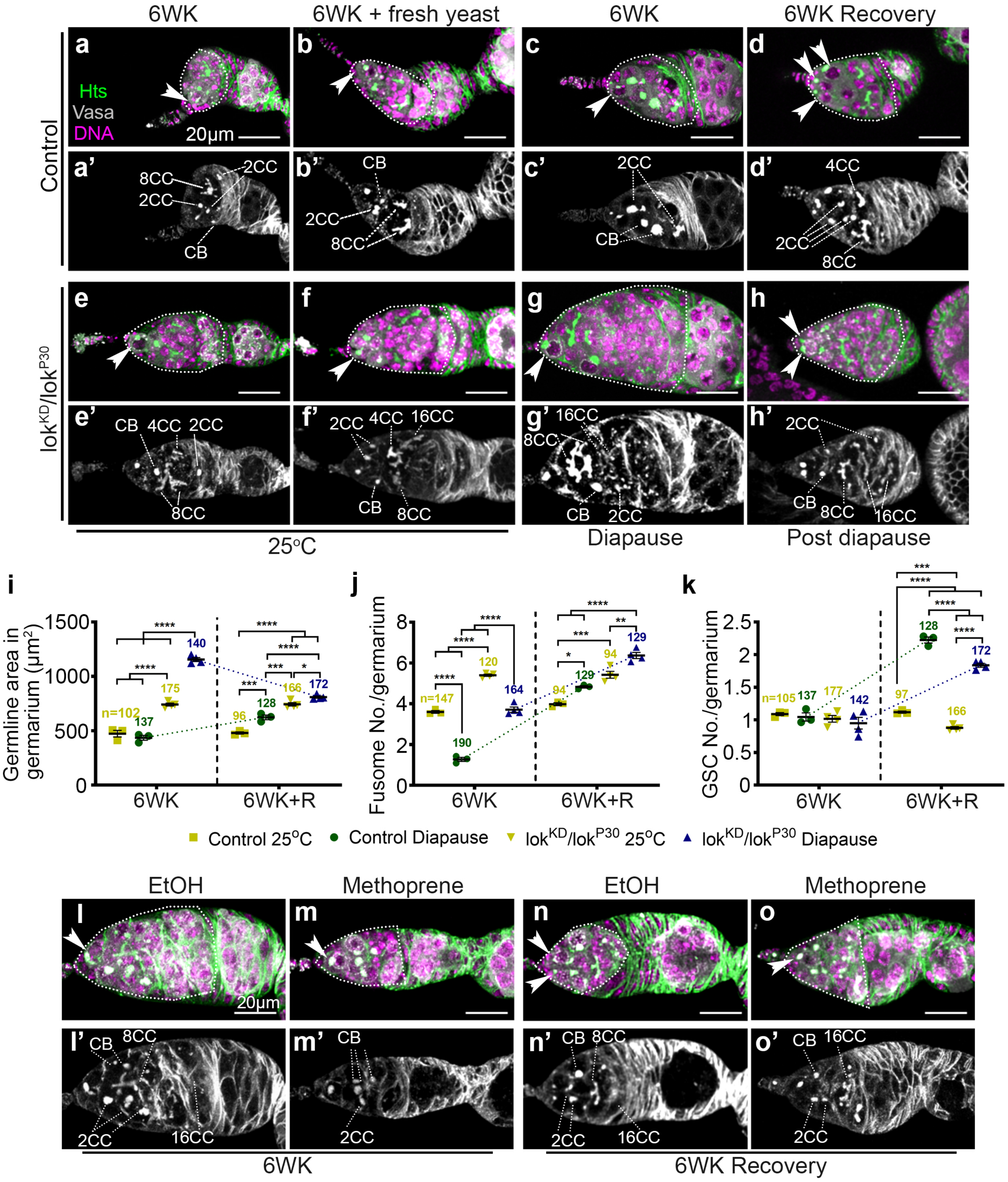
6WK
6WK + fresh yeast
6WK
6WK Recovery
Control
Hts
Vasa
DNA
20µm
a
b
c
d
a'
b'
c'
d'
8CC
2CC
CB
4CC
lokKD/lokP30
e
f
g
h
e'
f'
g'
h'
16CC
25°C
Diapause
Post diapause
i
j
k
Germline area in germarium (µm2)
Fusome No./germarium
GSC No./germarium
6WK
6WK+R
Control 25°C
Control Diapause
lokKD/lokP30 25°C
lokKD/lokP30 Diapause
EtOH
Methoprene
l
m
n
o
l'
m'
n'
o'
6WK Recovery

Supplementary Fig. 5: Chk2 DNA damage checkpoint maintains GSC diapause arrest.

a-h Representative images of germaria labeled with anti-Vasa (white), Hts (green), and Hoechst (magenta) from control (**a-d**) and lok-/- mutant (**e-h**) flies maintained for 6 weeks (WK) at 25°C (**a,b,e** and **f**) or in diapause (**c,d,g** and **h**) followed by recovery (**b,f,d** and **h**). **a'-h'** The Hts single channel alone allows identification of the cystoblasts (CB) and 2-, 4-, 8-, and 16-cell cysts (CC). Arrowheads indicate GSCs. Dotted lines outline germaria. **i-k** Quantification of germline area (**i -** *, p= 0.0264, ***, p<0.001; ****, p<0.0001), fusome number (**j -** *, p=0.0105; **, p=0.0019; ***, p=0.0001;****, p<0.0001) and GSC number (**k -** ***, p=0.0009; ****, p<0.0001) in the germarium from control and lok^{KD}/lok^{P30}. The control stain used for **a-d** is of Canton S strain. **l-o** Confocal micrographs of germaria from lok^{KD}/lok^{P30} in diapause and treated with either ethanol (EtOH, **l**) or methoprene for 6 weeks(WK) (**m**), followed by recovery from diapause (6WK Recovery, **r-s**) stained for Vasa (gray) and Hts(green) to identify the GSCs (marked with arrow-heads), Hoechst (gray). Germarium regions 1 and 2 are marked with dotted lines in **l-o**. **l'-o'** Hts channel in gray. n = number of germaria analyzed. Data are mean ± s.e.m. from at least 3 independent experiments. (**i-k**) 1way ANOVA and Tukey's multiple comparisons test. Scale bars are 20 μm.

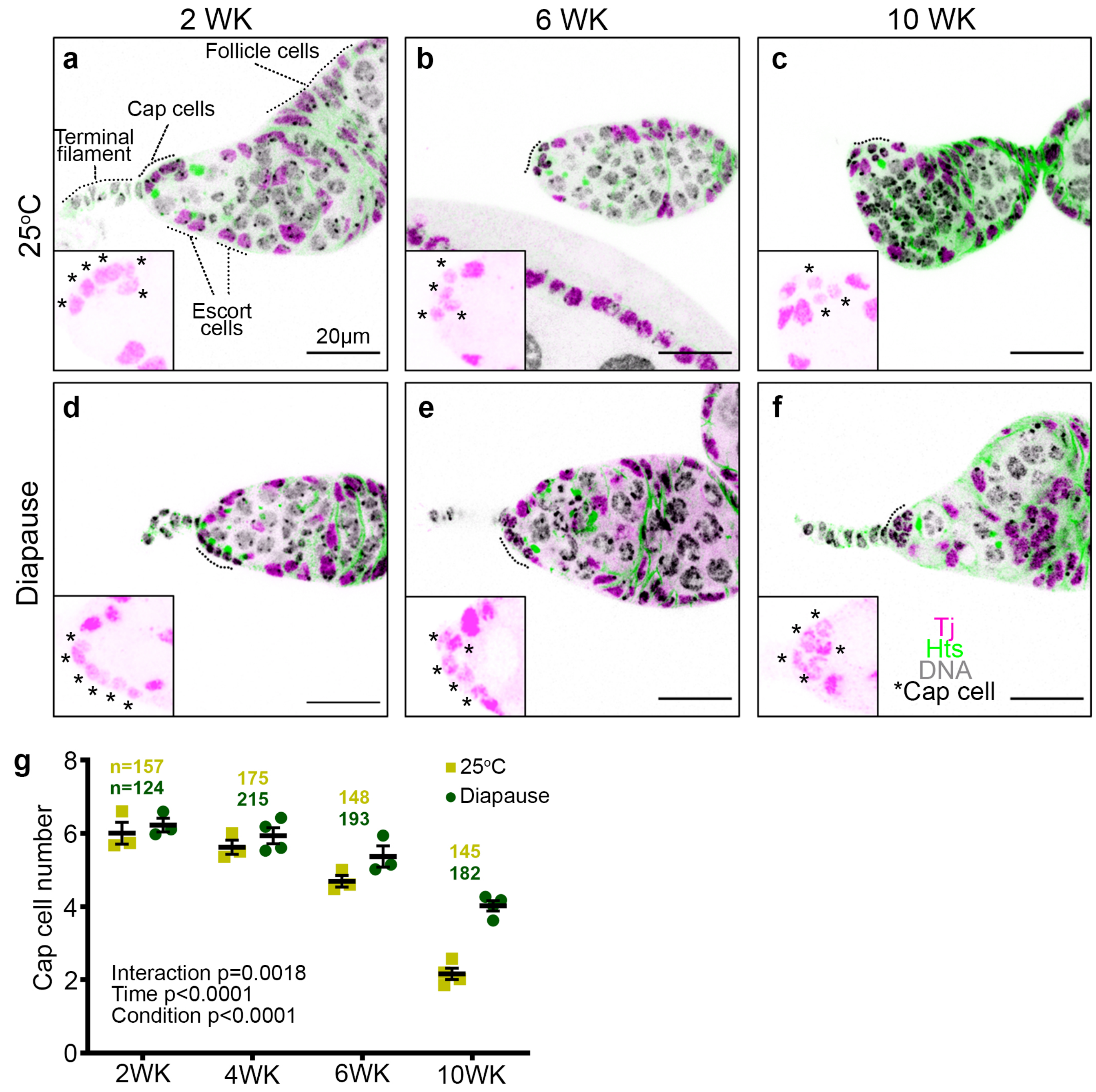

Supplementary Fig. 6: GSC niche preservation in diapause.

a-f Confocal micrographs of germaria stained for traffic jam (Tj, magenta) to identify the cap cells, Hts (green), and Hoechst (gray) from flies maintained at 25° (**a-c**) or in diapause conditions (**d-f**) for 2, 6, or 10 weeks (WK). Insets show the Tj channel. Asterisks mark cap cells, which are small, round, and label less intensely. **g** Quantification of cap cell numbers over time. n = number of germaria analyzed. Data are mean ± s.e.m. from at least 3 independent experiments and p values from 2way ANOVA are indicated. Scale bars are 20 µm.

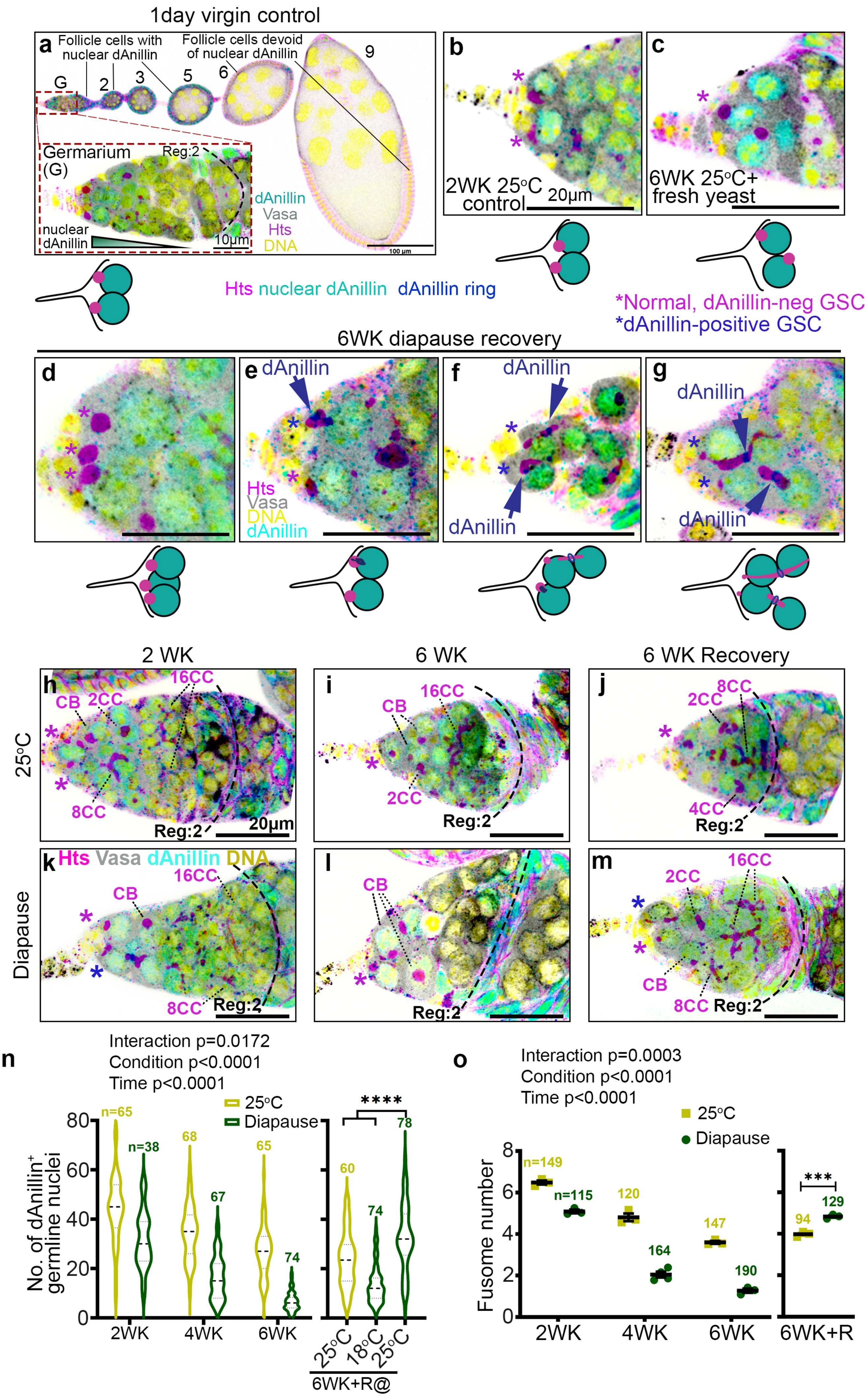
1day virgin control
a
Follicle cells with nuclear dAnillin
Follicle cells devoid of nuclear dAnillin
G 2 3 5 6 9
Germarium (G)
Reg:2
nuclear dAnillin
10μm
dAnillin
Vasa
Hts
DNA
100 μm
b
2WK 25°C control
20μm
c
6WK 25°C+ fresh yeast
Hts nuclear dAnillin dAnillin ring
*Normal, dAnillin-neg GSC
*dAnillin-positive GSC
6WK diapause recovery
d
e dAnillin
Hts
Vasa
DNA
dAnillin
f dAnillin
dAnillin
g dAnillin
dAnillin
2 WK
6 WK
6 WK Recovery
25°C
h CB 2CC 16CC 8CC Reg:2 20μm
i CB 16CC 2CC Reg:2
j 2CC 8CC 4CC Reg:2
Diapause
k Hts Vasa dAnillin DNA CB 16CC 8CC Reg:2
l CB Reg:2
m 2CC 16CC CB 8CC Reg:2
n
Interaction p=0.0172
Condition p<0.0001
Time p<0.0001
25°C
Diapause
No. of dAnillin+ germline nuclei
n=65 n=38 68 67 65 74 60 74 78
****
2WK 4WK 6WK
25°C 18°C 25°C
6WK+R@
o
Interaction p=0.0003
Condition p<0.0001
Time p<0.0001
25°C
Diapause
Fusome number
n=149 n=115 120 164 147 190 94 129
***
2WK 4WK 6WK 6WK+R

Supplementary Fig. 7: dAnillin staining in diapause and recovery.

a An ovariole from a virgin maintained for 1 day at 25°C showing loss of dAnillin (cyan) from follicle cell nuclei at stage 6, when they undergo the mitosis-to-endocycle transition. Inset shows high magnification of a germarium with nuclear dAnillin staining in the anterior cells, which declines and then is lost completely when the 16 cell-cyst enters the endocycle, concomitant with loss of the Hts-stained fusome (magenta). Hoechst staining of DNA (yellow) **b-g** Representative images of germaria stained for Hts (magenta), dAnillin (cyan), and Hoechst (yellow) from flies maintained for 2 weeks (WK) at 25°C (**b**), 6WK at 25°C with fresh yeast (**c**) or 6WK diapause and recovery (**d-g**). Corresponding illustrations highlight the normal GSCs (no dAnillin, dark blue, colocalisation with Hts) and GSCs having dAnillin colocalization with Hts. Asterisks indicate GSCs. Percentage of GSCs with Hts signal containing all or part of a dAnillin-ring are shown in **Supplementary Table 1**. dAnillin rings (blue arrowheads), which are normally found on ring canals, appear on some GSC spectrosomes or fusomes (**e-g**). **h-m** Representative images of germaria stained for Hts (magenta), dAnillin (cyan), and Hoechst (yellow) from flies maintained for 2 weeks (WK) at 25°C (**h**), 6WK at 25°C (**i**), 6WK at 25°C followed by supplementation with fresh yeast (**j**) or 2WK diapause (**k**), 6WK diapause (**l**), or 6WK diapause and recovery (**m**). **n** Quantification of germline cells with nuclear dAnillin. n= number of germaria analyzed. **o** Quantification of fusome number (indicative of developing cysts) in germaria from flies maintained in the indicated conditions. Data are mean ± s.e.m. from at least 3 independent experiments. ***, p=0.0007; ****, p<0.0001 (**n-o** 2way ANOVA before recovery and 1way ANOVA with Tukey's multiple comparisons test after recovery in **n;** unpaired two-tailed t-test after recovery in **o**). Scale bars are 20 µm or else mentioned within the image.

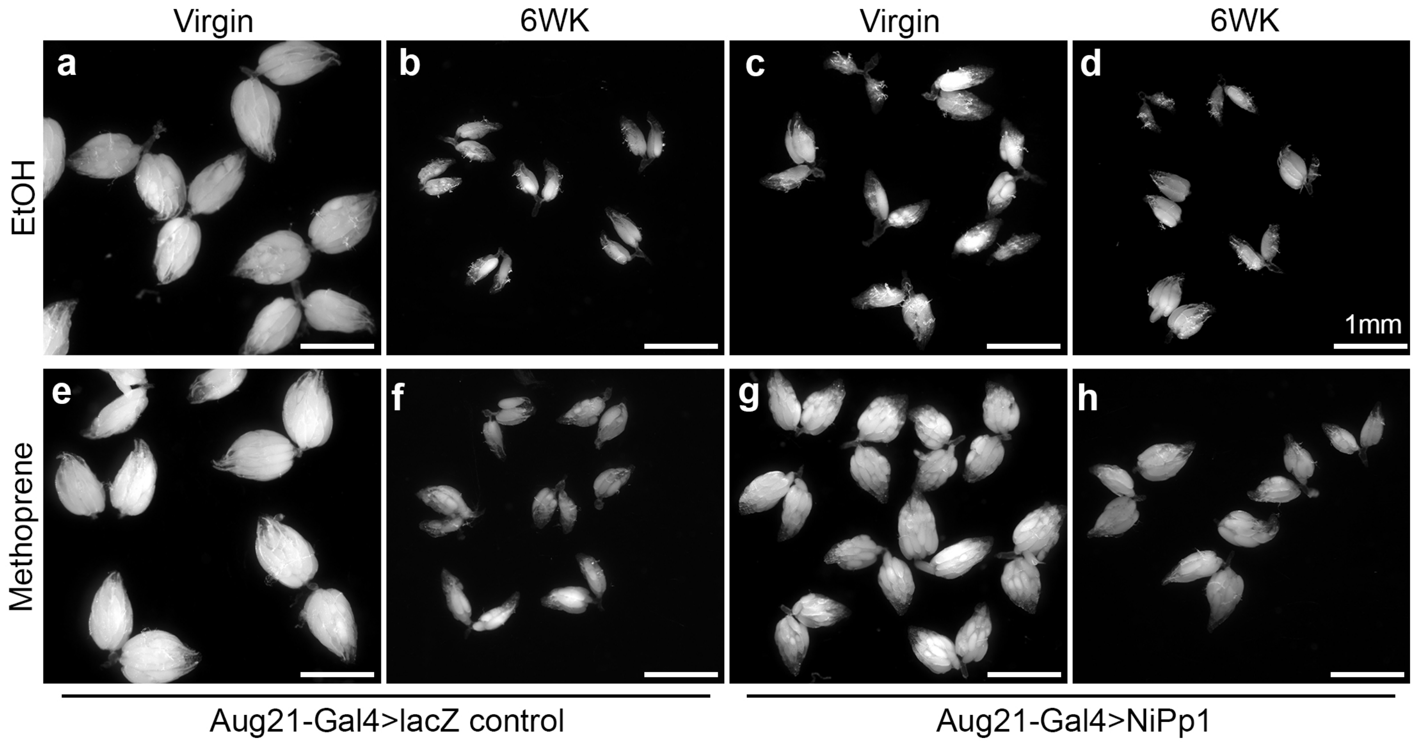

Supplementary Fig. 8: Effects of yolk deposition arrest in non-diapause conditions.

a-h Whole ovaries from young or aged control (**a**, **b**, **e** and **f**) flies and those with JH down regulation due to corpora allata ablation (**c**, **d**, **g** and **h**), either treated with ethanol as vehicle control (**a-d**) or methoprene (**e-h**), a JH analog. Scale bars are 1mm.

Supplementary Table 1: **Percentage of dAnillin^{+ve} GSCs at normal developmental condition and diapausing condition across different time period and recovery**

**Age in weeks**	**n**	**Average GSC No.**	**Average dAnillin^{+ve} GSCs**	**% dAnillin^{+ve} GSCs**
**<u>Control</u>**				
2WK	65	2.2	0.3	13.3
4WK	68	1.7	0.3	20.4
6WK	65	1.3	0.2	14.0
6WK + Recovery	60	1.2	0.3	27.1
**<u>Diapause</u>**				
2WK	38	2.1	0.6	30.7
4WK	67	1.6	0.8	51.6
6WK	74	1.2	0.5	42.6
6WK + 1day Recovery	74	1.4	0.8	60.1
6WK + 2day Recovery	78	2.3	1.0	41.5

Note - dAnillin^{+ve} = GSCs with Hts staining colocalizing all or part of dAnillin ring.

SUPPLEMENTARY METHODS

Wasp culturing and exposure

The larval endoparasitoid *Leptopilina heterotoma* (strain Lh14) used for the experiments were a kind gift from Giovanni Bosco. In order to culture the wasps, we allowed *D. virilis*, an immunocompromised strain of Drosophila, to lay eggs for at least 5 days in standard food bottles containing dry yeast. The flies were then replaced by adult Lh14 that subsequently parasitized developing *D. virilis* larvae. Bottles were supplemented with approximately 0.5 mL of 50% honey/water solution applied to the inside of the cotton plug. Newly eclosed wasps were transferred to vials containing Kimwipe supplemented with 1mL of the honey solution. Wasps aged 3–7 days old post-eclosion were used for all experiments and never reused.

To assay the effects of predator exposure on ovary development, we exposed five 3-day old virgin CS females to either 3 female Lh14 wasps or no wasps in a single vial. These flies were then incubated at 25°C for either 24 hours, 1 week or 2 weeks prior to dissection. The vials containing flies and/or wasps kept for 1 or 2 weeks were flipped to new food every other day to prevent desiccation.
