# Supplementary material for: Enhanced germline stem cell longevity in Drosophila diapause
Source: Nat Commun. 2022 Feb 7;13:711. doi: 10.1038/s41467-022-28347-z (PMC8821637; doi:10.1038/s41467-022-28347-z)
Supplement: Supplementary file 2 — Reporting Summary [file 41467_2022_28347_MOESM2_ESM.pdf]

nature portfolio

Corresponding author(s): Denise J Montell

Last updated by author(s): Dec 20, 2021

Reporting Summary

Nature Portfolio wishes to improve the reproducibility of the work that we publish. This form provides structure for consistency and transparency in reporting. For further information on Nature Portfolio policies, see our Editorial Policies and the Editorial Policy Checklist.

Statistics

For all statistical analyses, confirm that the following items are present in the figure legend, table legend, main text, or Methods section.

n/a	Confirmed	
☐	☒	The exact sample size (*n*) for each experimental group/condition, given as a discrete number and unit of measurement
☐	☒	A statement on whether measurements were taken from distinct samples or whether the same sample was measured repeatedly
☐	☒	The statistical test(s) used AND whether they are one- or two-sided<br>*Only common tests should be described solely by name; describe more complex techniques in the Methods section.*
☒	☐	A description of all covariates tested
☐	☒	A description of any assumptions or corrections, such as tests of normality and adjustment for multiple comparisons
☐	☒	A full description of the statistical parameters including central tendency (e.g. means) or other basic estimates (e.g. regression coefficient) AND variation (e.g. standard deviation) or associated estimates of uncertainty (e.g. confidence intervals)
☐	☒	For null hypothesis testing, the test statistic (e.g. F, t, r) with confidence intervals, effect sizes, degrees of freedom and P value noted<br>*Give P values as exact values whenever suitable.*
☒	☐	For Bayesian analysis, information on the choice of priors and Markov chain Monte Carlo settings
☒	☐	For hierarchical and complex designs, identification of the appropriate level for tests and full reporting of outcomes
☒	☐	Estimates of effect sizes (e.g. Cohen's d, Pearson's r), indicating how they were calculated

Our web collection on statistics for biologists contains articles on many of the points above.

Software and code

Policy information about availability of computer code

Data collection	Zeiss ZEN 2012 SP5 FP3 (black) release version 14 and ZEN 2.5 (blue edition) of microscope software.
Data analysis	Image J 1.53c-f, Imaris v8.3.0, Graphpad Prism 9, SigmaPlot 10, Adobe Photoshop CC, Adobe Illustrator CC

For manuscripts utilizing custom algorithms or software that are central to the research but not yet described in published literature, software must be made available to editors and reviewers. We strongly encourage code deposition in a community repository (e.g. GitHub). See the Nature Portfolio guidelines for submitting code & software for further information.

Data

Policy information about availability of data

All manuscripts must include a data availability statement. This statement should provide the following information, where applicable:

- Accession codes, unique identifiers, or web links for publicly available datasets
- A description of any restrictions on data availability
- For clinical datasets or third party data, please ensure that the statement adheres to our policy

Complete data is available in the main article, supplementary materials and in the source data files. All the raw data supporting the findings of this study are available from the corresponding authors upon reasonable request.

Field-specific reporting

Please select the one below that is the best fit for your research. If you are not sure, read the appropriate sections before making your selection.

☒ Life sciences ☐ Behavioural & social sciences ☐ Ecological, evolutionary & environmental sciences

For a reference copy of the document with all sections, see nature.com/documents/nr-reporting-summary-flat.pdf

Life sciences study design

All studies must disclose on these points even when the disclosure is negative.

Sample size	Sample size was not statistically determined but all experiments were carried out using at least 10 animals per condition. Also the experiments were conducted and followed for different time periods as well as in multiple replicates with minimum of 3 times for reproducibility which from experience is sufficient to give true representation. Variability between the replicates were not dramatically changed with in each group and time period analyzed.
Data exclusions	No data were excluded from the analyses.
Replication	Experiments were repeated at least 3 times for reproducibility and were successful.
Randomization	Experimental groups were formed based on the genotype or based on treatments in case of same genotypes.<br>All the samples prepared, imaged or analyzed were randomly selected without any bias.
Blinding	Investigators were not blinded since the experiments were carried out over progressing aging conditions and different treatments with multiple controls. Also the different temperature set up is easily detectable and blinding is not standard in the field.

Reporting for specific materials, systems and methods

We require information from authors about some types of materials, experimental systems and methods used in many studies. Here, indicate whether each material, system or method listed is relevant to your study. If you are not sure if a list item applies to your research, read the appropriate section before selecting a response.

Materials & experimental systems

n/a	Involved in the study	
☐	☒	Antibodies
☒	☐	Eukaryotic cell lines
☒	☐	Palaeontology and archaeology
☐	☒	Animals and other organisms
☒	☐	Human research participants
☒	☐	Clinical data
☒	☐	Dual use research of concern

Methods

n/a	Involved in the study	
☒	☐	ChIP-seq
☒	☐	Flow cytometry
☒	☐	MRI-based neuroimaging

Antibodies

Antibodies used	The antibodies used in this study are rat anti-Vasa (anti-vasa; DSHB; 1:20), mouse anti-Hts(1B1; DSHB; 1:20), mouse anti-H2AvD (UNC93-5.2.1; DSHB; 1:20); guinea pig anti-traffic jam (Tj) (gift from Dorothea Godt; 1:5000); rabbit anti-pH3(Cell Signaling Tech, #9701, Lot. 16; 1:200); rabbit anti-pMAD/anti-Smad (Abcam, ab52903, Lot. GR3194559-4; 1:400); mouse anti-Dpp (R&D SYSTEMS, MAB159, Lot. FRW022006A; 1:200); rabbit anti-dAnillin8-8(Gift from Christine Field; 1:500) and rabbit anti-ACTIVE Caspase-3 (Promega, G748A, Lot. 0000237765; 1:500).
Validation	anti-Vasa - please see Yan Song and Bingwei Lu, Genes Dev 2011 and https://dshb.biology.uiowa.edu/anti-vasa<br>anti-Hts - please see Rachel T Cox and Allan C Spradling, Development 2003 and https://dshb.biology.uiowa.edu/1B1<br>anti-H2AvD - please see Cathleen M Lake et al., G3(Bethesda) 2013 and https://dshb.biology.uiowa.edu/UNC93-5-2-1<br>anti-traffic jam - please see Trupti Panchal et al., PLoS Genet 2017<br>anti-pH3 - please see Natalie A. Dye et al., Development 2017 and https://www.cellsignal.com/products/primary-antibodies/phospho-histone-h3-ser10-antibody/9701<br>anti-pMAD/anti-Smad - please see https://www.abcam.com/smad3-phospho-s423--s425-antibody-ep823y-ab52903.html?productWallTab=ShowAll<br>anti-Dpp - please see Yu-Han Su et al., Development 2018 and https://www.rndsystems.com/products/drosophila-decapentaplegic-dpp-antibody-146609_mab159<br>anti-dAnillin - please see Christine M Field and Bruce M Alberts, J Cell Biol 1995<br>anti-ACTIVE Caspase-3 - please see https://www.promega.com/-/media/files/resources/protocols/product-information-sheets/g/

antiactive-caspase-3-pab-protocol.pdf?la=en

Animals and other organisms

Policy information about studies involving animals; ARRIVE guidelines recommended for reporting animal research

Laboratory animals	Study involved Drosophila melanogaster, Drosophila virilis (gift from Giovanni Bosco) and Leptopilina heterotoma Lh14 strain (gift from Giovanni Bosco). The Drosophila melanogater strains were from Bloomington Drosophila Stock Center (BDSC) - Canton-S (CS) (#64349), Ubi-FRT-STOP-FRT-nEGFP (#32251), hs-FLP (#55815), Aug21-GAL4 (#30137), UAS-HA-NiPp1 (#23711); p53[5A-1-4] (#6815); BamΔ86 (#5427); bam deficiency (#27401); tub-mito-roGFP2-Orp1 (#67673); UASp-PAGFP-alphaTub84B (#32075); nos-GAL4 vp16 (gift from Jecelyn McDonald); lok-KD and lok-P30 (gift from Pamela Geyer); P53R-GFP NLS biosensor (gift from John M Abrams). Female virgin flies are used in experiments with different age/time period as mentioned for different experiments.
Wild animals	Study did not involve wild animals.
Field-collected samples	Study did not involve samples collected from the field.
Ethics oversight	Drosophila and wasp strains were obtained and reared according to standard protocols and institutional regulations from the University of California Santa Barbara.

Note that full information on the approval of the study protocol must also be provided in the manuscript.
